# Adenosinergic Signalling in Cervical Cancer Microenvironment

**DOI:** 10.1017/erm.2024.30

**Published:** 2025-01-07

**Authors:** Isabele Cristiana Iser, Ana Paula Santin Bertoni, Liziane Raquel Beckenkamp, Marcia Edilaine Lopes Consolaro, Silvya Stuchi Maria-Engler, Marcia Rosângela Wink

**Affiliations:** 1 Department of Basics Health Sciences and Laboratory of Cell Biology, Federal University of Health Sciences of Porto Alegre, Porto Alegre, RS, Brazil; 2 Department of Clinical Analysis and Biomedicine, Division of Clinical Cytology, State University of Maringá, Maringá, PR, Brazil; 3 Department of Clinical Chemistry and Toxicology, School of Pharmaceutical Sciences, University of São Paulo, São Paulo, SP, Brazil

**Keywords:** CD73, *NT5E*, adenosine, adenosine kinase, cervical cancer, methylation

## Abstract

Despite the emergence of the first human papillomavirus vaccine, the incidence of cervical cancer is still responsible for more than 350,000 deaths yearly. Over the past decade, ecto-5′-nucleotidase (CD73/5′-NT) and extracellular adenosine (ADO) signalling has been the subject of many investigations to target cancer progression. In general, the adenosinergic axis has been linked to tumourigenic effects. However, CD73 can play contradictory effects, probably dependent on the tumour type, tumour microenvironment and tumour stage, thus being in some circumstances, inversely related to tumour progression. We herein reviewed the pathophysiological function of CD73 in cervical cancer and performed *in silico* analysis of the main components of the adenosinergic signalling in human tissues of cervical cancer compared to non-tumour cervix tissue. Our data showed that the *NT5E* gene, that encoded CD73, is hypermethylated, leading to a decreased CD73 expression in cervical cancer cells compared to normal cells. Consequently, the high availability of ADO cytoplasmatic/extracellular leads to its conversion to AMP by ADK, culminating in global hypermethylation. Therefore, epigenetic modulation may reveal a new role for CD73 in cervical cancer.

## Introduction

Although cervical cancer is one of the most preventable and treatable forms of cancer, it remains the fourth most common cancer in women worldwide, with estimated 350,000 deaths yearly (Ref. 1). Almost all cervical cancer cases are linked to persistent infection with high-risk human papillomaviruses (HPVs) (Ref. 1) and the treatment depends on the disease extent, which may include surgery and/or a combination of chemotherapy and radiotherapy (chemoradiation). Meanwhile, for women with metastatic or recurrent disease, the overall prognosis remains poor (Ref. 2).

Accumulating evidence from the literature suggests the involvement of purinergic signalling, particularly the adenosinergic axis, in cancer progression (Refs 3–5), affecting mechanisms such as cell proliferation, angiogenesis, immune responses, drug resistance and cell death (Ref. 6, 7). Molecules such as adenosine triphosphate (ATP) and adenosine (ADO) are present in the tumour microenvironment (TME) and directly affect tumour cell growth, immune cell function and tumour dissemination. However, if the extracellular ADO and/or ATP will be beneficial or malefic for the tumour growth depends on their concentration, rate of degradation by enzymes and the panel of receptors expressed by the tumour cells and other cells present in the TME (Ref. 8).

In this review, we will have a special focus on the role of ecto-5′-nucleotidase (CD73/5′-NT)/ADO in cervical cancer, describing the canonical and non-canonical pathways capable of promoting the accumulation of ADO in the TME. To deepen our discussion, we performed *in silico* investigation, showing a significant downregulation of CD73 in human cervical cancer samples in comparison to non-tumour tissue in most datasets analysed. We also showed that the reduced CD73 expression in cervical cancer might be due to hypermethylation in the *NT5E* gene.

## Adenosine is produced by canonical and non-canonical pathways

Purinergic signalling is characterized by the activity of ectoenzymes called ectonucleotidases, which control the levels of nucleotides and nucleosides present in the extracellular microenvironment (Ref. 9). There are different families of ectonucleotidases described, with varied hydrolysis ability and affinity potential for purines (ATP, adenosine diphosphate (ADP), adenosine monophosphate (AMP) and ADO) and pyrimidines (uridine-5′-triphosphate (UTP) and uridine diphosphate (UDP)) (Ref. 9). Among these families, we can mention E-NTPDases (ectonucleoside triphosphate-diphosphohydrolases), CD73, E-NPPs (ectonucleotide pyrophosphatase/phosphodiesterases), alkaline phosphatase and adenosine deaminase (ADA). These enzymes, alone or in combination, will exert an essential role in regulating purinergic signal transmission through hydrolysis of nucleotides/nucleosides, thereby controlling persistence, conclusion or restart of the signalling. CD73, specifically, is an ectoenzyme encoded by the *NT5E* gene, able to catabolize AMP into ADO at the extracellular level (Ref. 9).

**Figure 1.**
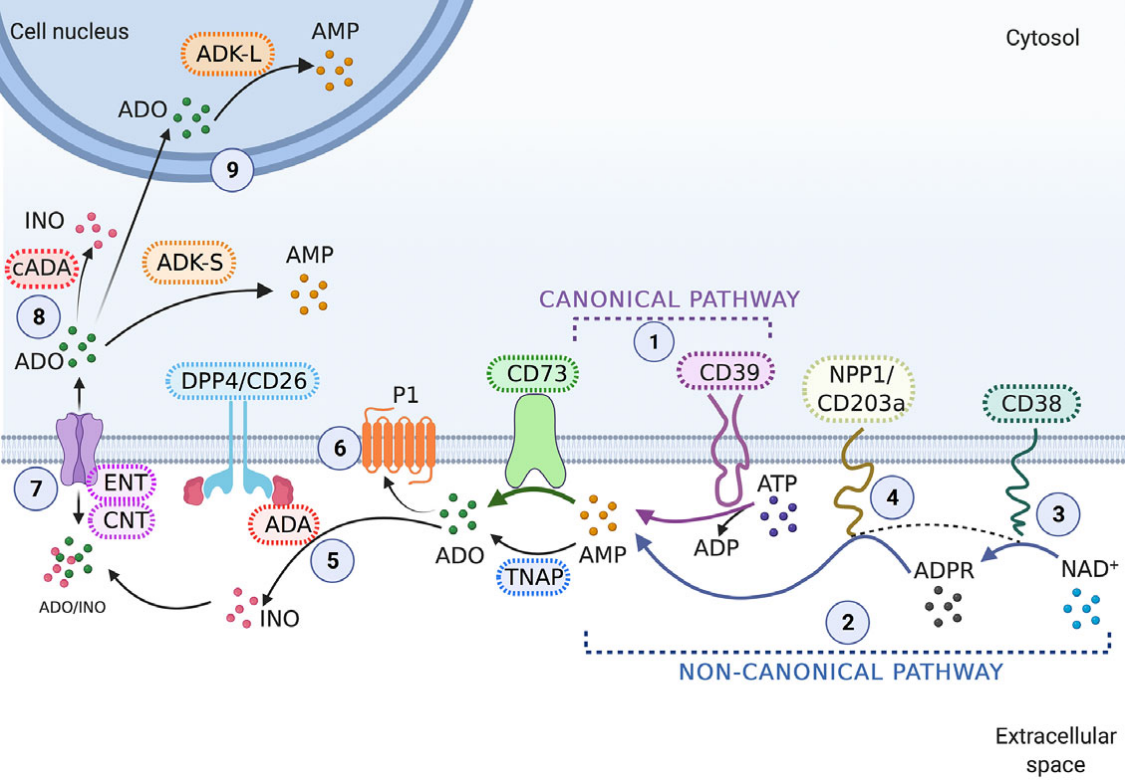
Cellular pathways regulating adenosinergic signalling. (1) The canonical pathway is the main way of extracellular ADO production, in which extracellular ATP is first hydrolyzed by the NTPDase1/CD39 to AMP. The AMP molecules can be further hydrolyzed by the CD73/5′-NT, thereby generating ADO. (2) The non-canonical cascade is an alternative ADO-generating pathway, which allows the production of ADO independent of CD39. (3) First, NAD+ is hydrolyzed by CD38, generating adenosine diphosphate ribose (ADPR), (4) which, in turn, is hydrolyzed by NPP1/CD203a, producing AMP. CD203a can also hydrolyze NAD+ or ATP directly generating AMP which can be hydrolyzed by CD73 to ADO. (5) Extracellular ADO can either be catabolized to inosine (INO) by extracellular adenosine deaminase (ADA), (6) activate the type 1 purinergic (P1) receptors (A1, A2a, A2b and A3) or (7) be transported into cells by equilibrative (ENT1/2) and/or concentrative (CNT1/2) nucleoside transporters. (8) Once in the cytosol, ADO can be degraded to inosine (INO) by cytosolic adenosine deaminase (ADA) or (9) re-phosphorylated to AMP by adenosine kinase (ADK-S in the cytoplasm and ADK-L in the nucleus).

**Figure 2.**
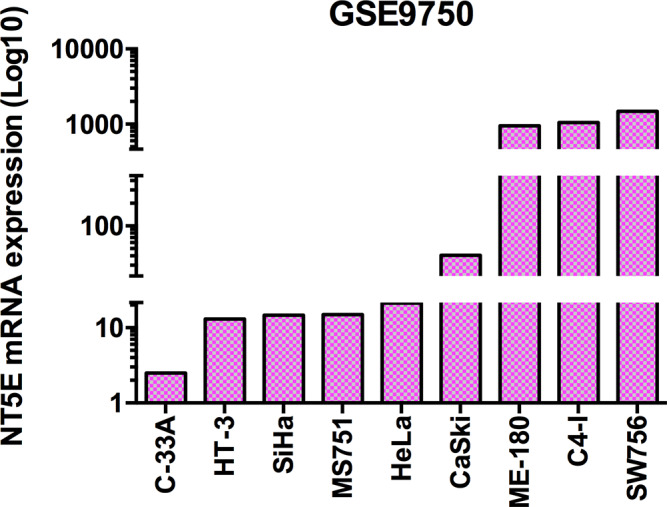
Profile of CD73 (*NT5E*) expression on cervical cancer cell lines from the GSE9750 dataset.

**Figure 3.**
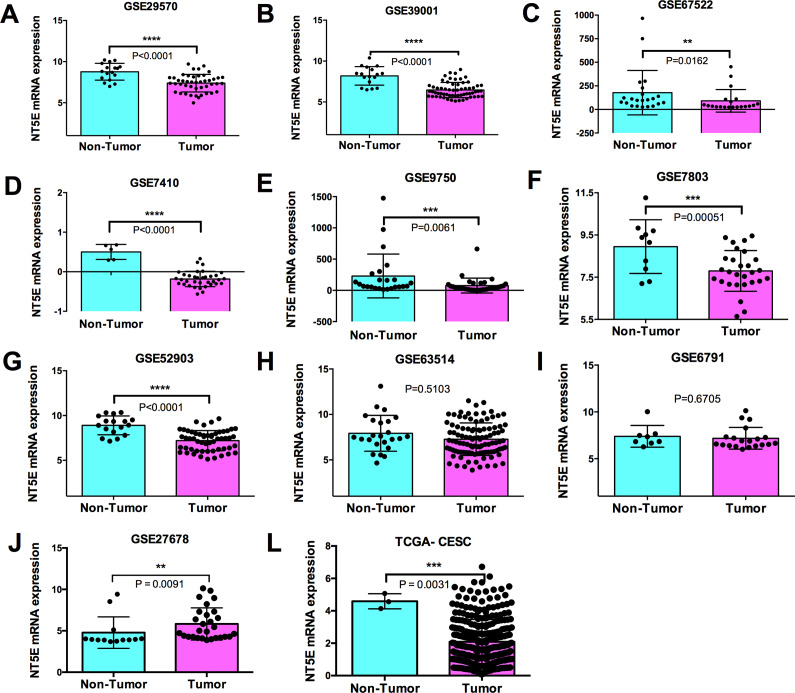
Expression of CD73 (*NT5E*) in cervical samples and non-tumour tissues. Relative expression of CD73 mRNA in cervical cancer and non-tumour tissues is shown in scatter dot-plots from GSE29750 (A), GSE39001 (B), GSE67522 (C), GSE7410 (D), GSE9750 (E), GSE7803 (F), GSE52903 (G), GSE63514 (H), GSE6791 (I), GSE27678 (J) and TCGA-CESC (K) datasets. Data presented as mean ± standard deviation and statistical significances (*P* values) between groups were determined by the Mann–Whitney U-test or two-tailed Student’s t-test.

**Figure 4.**
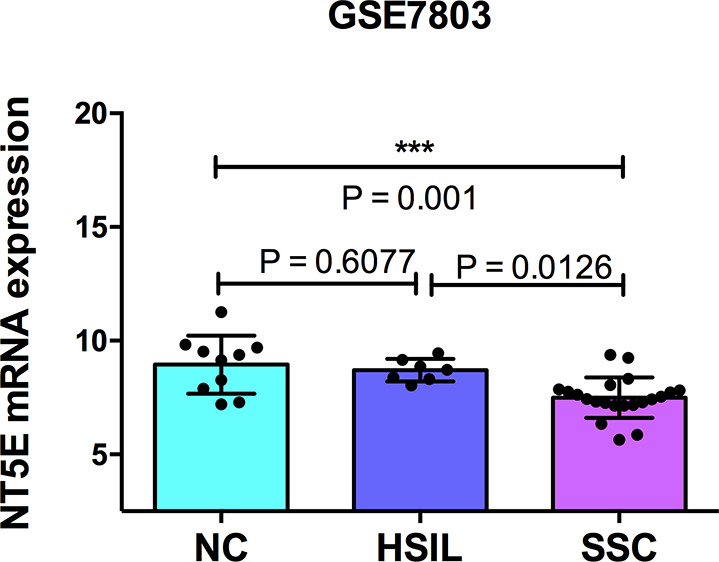
Differential CD73 (*NT5E*) expression in normal cervix, preinvasive and invasive cervical lesions. Each dot in the scatter plot represents the individual sample from GSE7308 stratified as normal cervix (NC; *n* = 10), high-grade squamous intraepithelial lesions (HSIL; *n* = 7) and invasive squamous cell carcinomas of the cervix (NSIL; *n* = 21). Data presented as mean ± standard deviation and statistical significance (*P* values) between groups were determined by two-tailed Student’s t-test.

**Figure 5.**
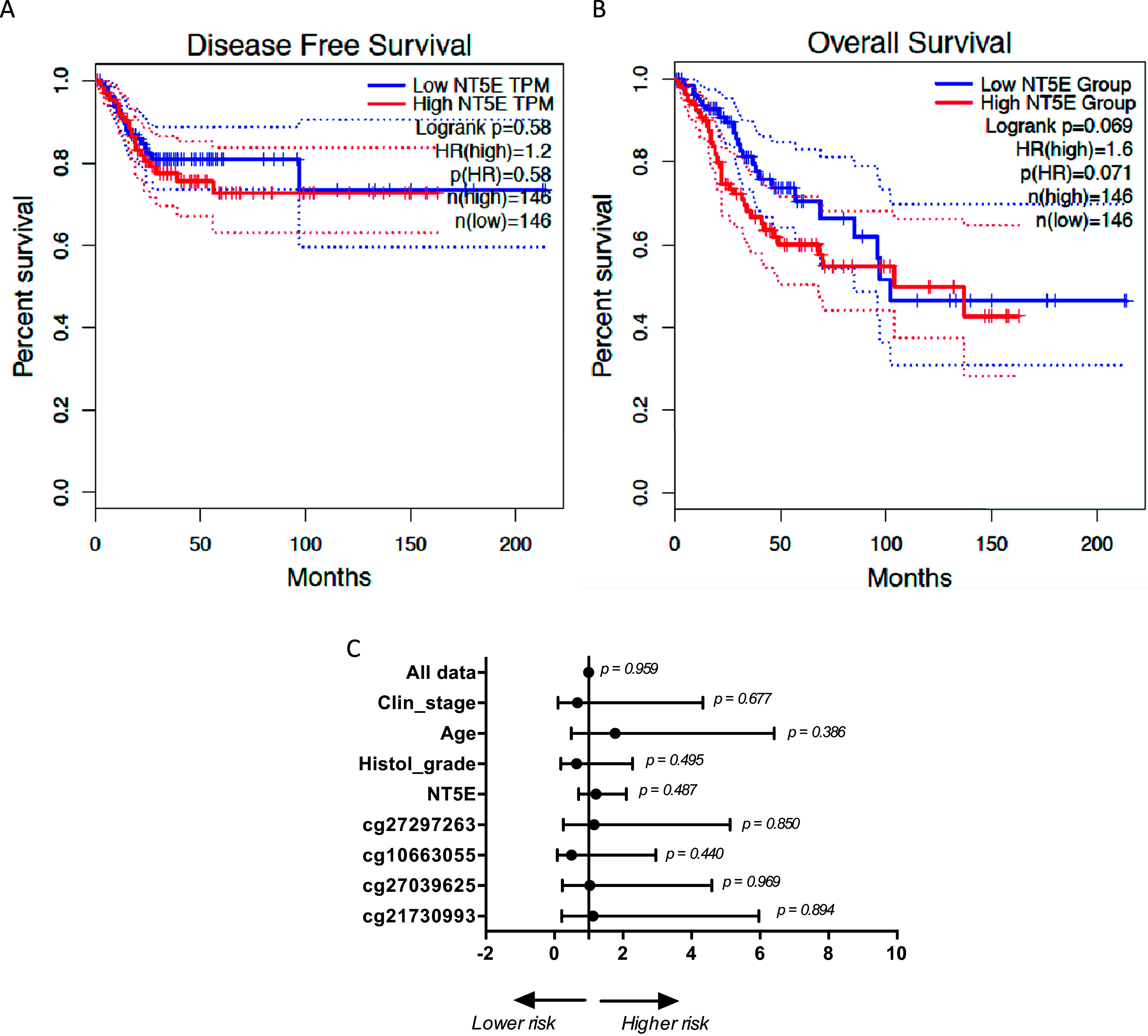
Estimated overall survival curve for CD73 (*NT5E*). (A) Kaplan–Meier curves of disease-free survival and (B) overall survival of patients with cervical cancer based on CD73 mRNA expression (low (*n* = 146) versus high (*n* = 146) transcripts of per million (TPM); hazard ratio (HR) = 1.6). The solid lines indicate the survival curve, and the dotted lines indicate the 95% confidence interval. Overall survival analyses were performed using the GEPIA online platform (http://gepia.cancer-pku.cn/). (C) Forest plot of the multivariate Cox regression analysis showing the risk score for clinical factors and *NT5E* expression.

**Figure 6.**
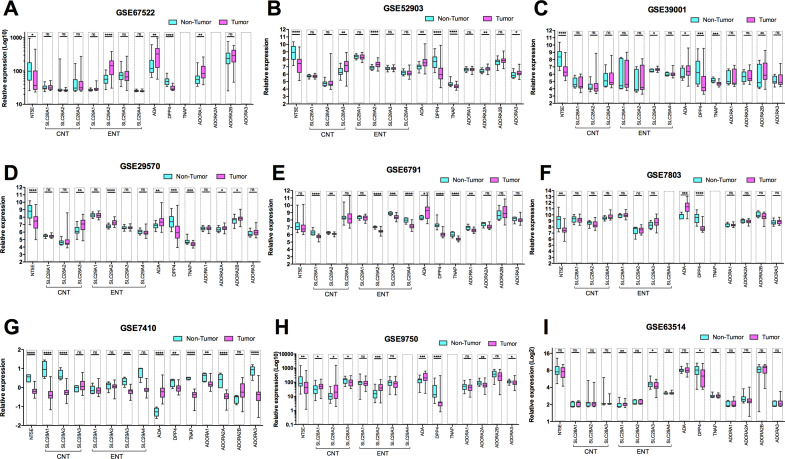
Gene expression modulation of adenosinergic-related components in normal cervix and cervical cancer. The boxplots show the expression levels of CD73 (*NT5E*), concentrative nucleoside transporters (CNTs: SLC28A1, SLC28A1 and SLC28A1), equilibrative nucleoside transporters (ENTs: SLC29A1, SLC29A2, SLC29A3 and SLC29A4), adenosine deaminase (ADA), the surface glycoprotein CD26 (DPP4), tissue-nonspecific alkaline phosphatase (TNAP) and adenosine receptors (ADORA1, ADORA2A, ADORA2B and ADORA3) genes. The relative expressions were collected from the GEO database through microarray data using (A) GSE67522, (B) GSE52903, (C) GSE39001, (D) GSE29570, (E) GSE6791, (F) GSE7803, (G) GSE7410, (H) GSE9750 and (I) GSE63514 datasets. Data were expressed as mean, and the upper and lower whiskers represent the maximum and minimum values of gene expression. The P value was considered non-significant (ns) if equal to or less than 0.050 and statistically significant when *P = 0.049–0.01, **P = 0.01–0.001, ***P < 0.0001, ****P < 0.00001.

Extracellular nucleotides and nucleosides exert their effects through interactions with specific membrane receptors, known as purinergic receptors (Ref. 10). The P2-type receptors are subdivided into ionotropic (P2X 1–7) and metabotropic (P2Y 1, 2, 4, 6, 11–14) receptors and exhibit different ranges of responsiveness to ATP, UTP and its diphosphate analogues (Ref. 10). On the other hand, the P1-type receptors (A1, A2A, A2B and A3) are responsive mainly to ADO (Figure 1 – (6)) (Ref. 10).

Purinergic signalling is an important mechanism used by cells to control their internal events and interact with the external environment (Ref. 11). ATP is released from different cell types and is considered a key component of the purinergic system mainly because the sequential removal of its phosphate groups results in the formation of the nucleoside ADO (Ref. 11). Extracellular ATP and ADO, as well as purinergic enzymes, receptors and transporters, are part of this signalling system, which links cellular metabolism to other cellular processes, including proliferation, differentiation and cell death (Ref. 11).

The canonical pathway of ADO production involves E-NTPDase 1 (NTPDase1/CD39), which hydrolyzes ATP, producing AMP. Under the action of CD73, AMP is then hydrolyzed to ADO, completing the adenosinergic loop (Figure 1 – (1)) (Refs 12, 13). The non-canonical pathway is an alternative adenosine production route, independent of CD39 (Figure 1 – (2)). This pathway involves the production of ADP-ribose from NAD+ through CD38 (Figure 1 – (3)), which is then processed to AMP by ENPP1/CD203a (Figure 1 – (4)) (Refs 12, 14). Finally, the AMP is metabolized to ADO by CD73, which, in turn, acts as a link between the canonical and non-canonical pathways (Figure 1) (Ref. 12). The extracellular catalytic activity of CD73 can be performed by a membrane-bound form of CD73 or a soluble form derived from the glycosylphosphatidylinositol (GPI)-anchored CD73 by cleavage (Ref. 15). Although the main focus of this work is to explore the role of CD73 in cervical cancer, we must also mention that the ability to metabolize AMP is not an exclusiveness of CD73. Other enzymes, such as tissue-specific alkaline phosphatases and tissue-non-specific alkaline phosphatases (TNAPs), can also hydrolyze AMP to ADO (Figure 1) (Ref. 16).

**Figure 7.**
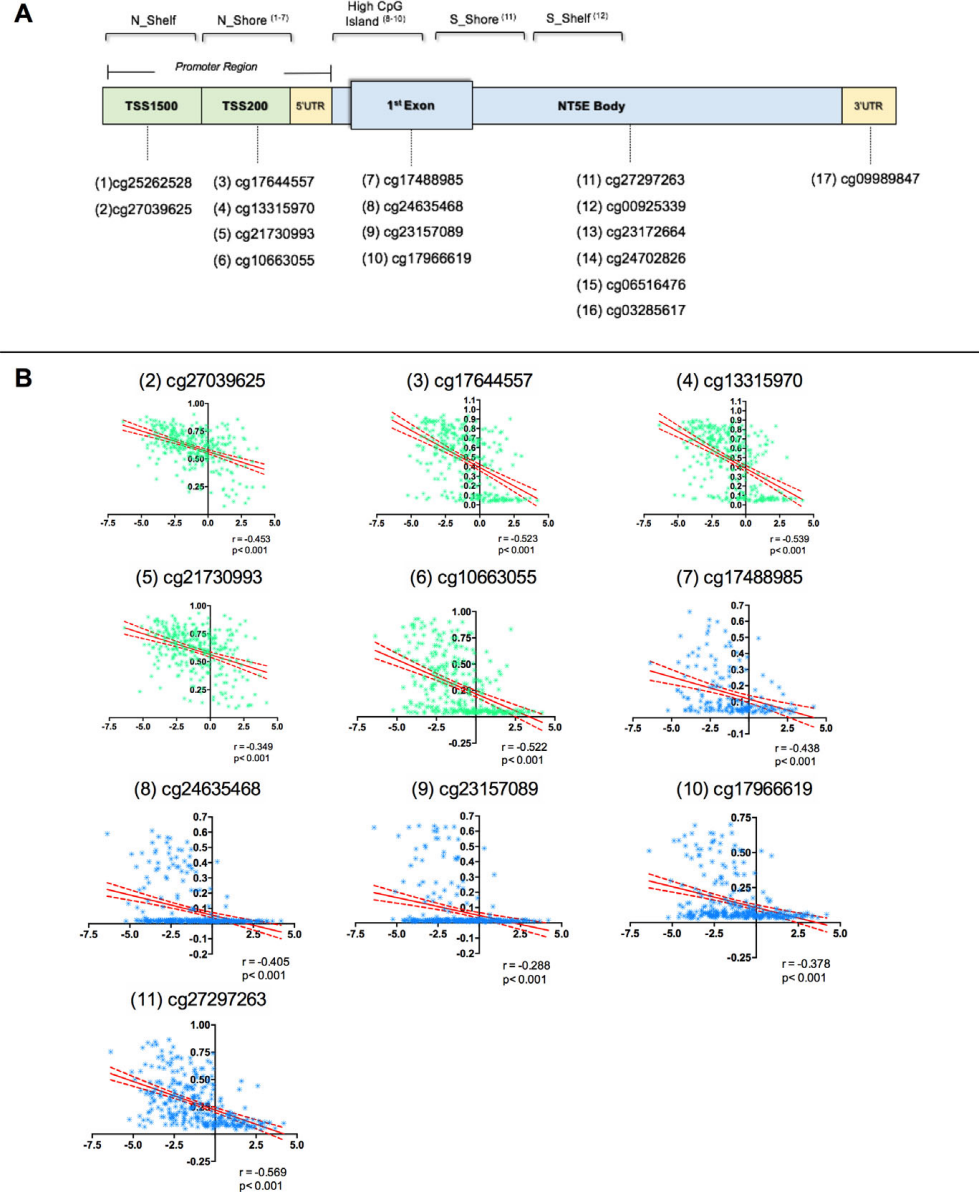
Correlation between CD73 (*NT5E*) mRNA expression and methylation levels from TCGA-CESC. (A) Schematic presentation of cytosine-phosphate-guanine (CpG) site distribution in the CD73 promoter, 1st exon and body. (B) Scatter plot showing the Pearson’s correlation coefficient (*r*) between methylation levels (*Y*-axis) and relative expression of CD73 (*X*-axis) in samples of cervical cancer from TCGA. The solid lines indicate a linear fit, and the dotted lines indicate the 95% confidence interval for the correlation.

**Figure 8.**
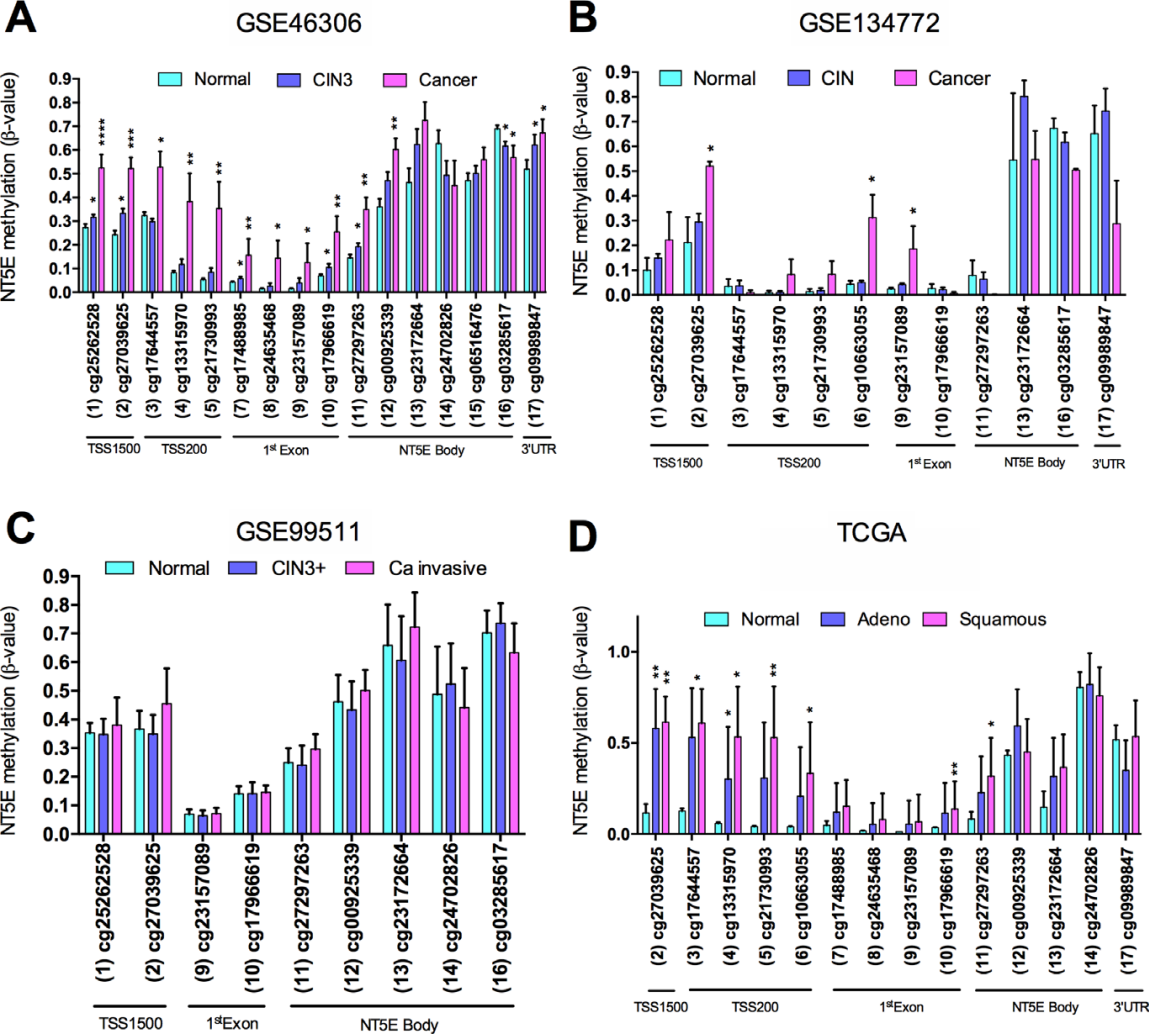
DNA methylation profile in normal cervix and cervical cancer. Bars show the DNA methylation β-values (*Y*-axis) from (A) GSE46306, (B) GSE134772, (C) GSE99511 datasets and from (D) The Cancer Genome Atlas (TCGA) cervical squamous cell carcinoma and endocervical adenocarcinoma database. Data represent mean ± standard deviation. Adeno: adenocarcinoma; CIN: cervical intraepithelial neoplasia; CIN3+: CIN grade 3; TSS: transcription start sites; TSS200: 0–200 nucleotides upstream of the TSS; TSS1500: 200–1500 nucleotides upstream of the TSS; 3′-UTR: three prime untranslated region; *P = 0.05–0.01, **P = 0.01–0.001, ***P < 0.0001.

**Figure 9.**
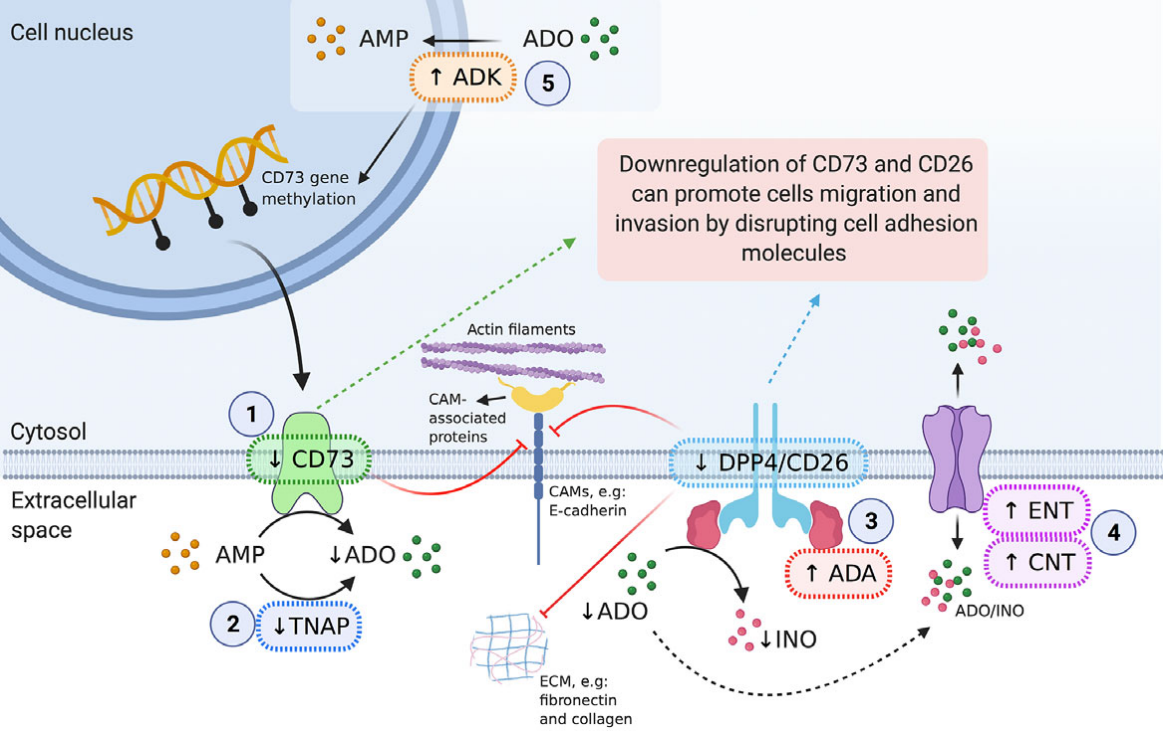
Overview of adenosinergic regulation in cervical cancer. CD73 (*NT5E*) is downregulated in cervical cancer, when compared to normal tissues, which can be explained by methylation in the NT5E CpG island. The cell-to-cell epigenetic heterogeneity could also justify the variability in scientific literature regarding CD73 expression in cervical cancer. (1) In extracellular space, the AMP can be hydrolyzed by CD73 (also known as 5′-nucleotidase) to produce extracellular adenosine ADO. However, the reduced levels of CD73 in cervical cancer, consequently, will generate less ADO in extracellular space. (2) Lower levels of extracellular ADO could be also a consequence of a reduced expression of tissue-non-specific alkaline phosphatase (TNAP), which is able to convert AMP in ADO. The ADO can be (3) metabolized to inosine (INO) by ecto-adenosine deaminase (ADA), (4) transported into cells via equilibrative or concentrative nucleoside transporters (ENTs and CNTs, respectively) or (5) phosphorylated to AMP via adenosine kinase (ADK). Adenosine kinase is also involved with DNA methylation, thus being able to modulate CD73 expression levels. The expression of CD26 (DPP4) is decreased in cervical cancer; consequently, the ADA activity is impaired since CD26 acts as the main cellular binding protein for anchorage of ADA. CD26 is known as a tumour suppressor molecule, being that decreased CD26 expression has been correlated to a higher invasive potential. This can be explained mainly because CD26 interacts with ECM proteins, such as collagen and fibronectin, modulating the extracellular environment and upregulating cell adhesion molecules (CAMs), such as E-cadherin and β-catenin, indicating that this protein may act as an adhesion molecule. In the same way, CD73 and ADO can upregulate CAMs (e.g.: E-cadherin and β-catenin) at the cell membrane, leading to maintenance of cell–cell adhesions and inhibiting cell migration and invasion.

At the end of the purinergic signalling, ADO can be degraded to inosine by ADA (Figure 1 – (5)) or be uptaken into the intracellular environment through both equilibrative (ENT1/2) and concentrative (CNT1/2) nucleoside transporters (Figure 1 – (7)) (Ref. 17). In the intracellular compartment, ADO can be degraded to INO by cytosolic ADA (Figure 1 – (8)) or re-phosphorylated to AMP by adenosine kinase (ADK) (Figure 1 – (9)). The ADK can be found in a cytoplasmic (ADK-S) and a nuclear (ADK-L) isoform (Figure 1 – (9)), but both drive the phosphorylation of ADO to form AMP (Ref. 18). The cytoplasmic short isoform ADK-S provides the main metabolic way of ADO clearance under normal conditions and, consequently, regulates intra- and extracellular levels of ADO by phosphorylating ADO in AMP (Ref. 19). In contrast, the long isoform ADK-L acts as a regulator of DNA methylation. The activity of ADK-L decreases the concentration of ADO, favouring the S-adenosylmethionine (SAM)-dependent transmethylation pathway, which drives DNA and histone methylation. Thereby, high levels of ADK-L are associated with increased DNA methylation in the nucleus and, consequently, contribute to the regulation of gene expression (Refs 18, 20, 21). An overview of this signalling cascade is summarized in Figure 1.

## Adenosinergic signalling in cancer

The CD73/5′-NT hydrolysis activity leads to ADO production, which is related to an immunosuppressed environment, commonly associated with cancer development and progression (Ref. 22). The importance of the CD73/ADO pathway in cancer has been extensively demonstrated (Refs 6, 23, 24). The ADO generated by the action of the CD73 enzyme can lead to decreasing activity of several immune cell lineages, including CD4 and CD8 T cells, natural killer cells and antigen-presenting cells. On the other side, it promotes the enhancement of the function of Treg cells, MDSCs and tumour-associated macrophages (TAM), further reinforcing immunosuppression (Refs 25, 26). Furthermore, extracellular ADO regulates the growth and dissemination of cancer by direct actions in tumour cell migration, invasion, metastasis and proliferation (Refs 25, 27). Also, it stimulates the formation of cancer stem cells, which have a direct role in tumour heterogeneity and resistance to therapy (Ref. 28).

In human breast cancer cells, the treatment with adenosine 5′-(α,β-methylene)diphosphate (APCP), a competitive CD73 inhibitor, decreased proliferation rate and cell viability induced by CD73 overexpression (Ref. 29). In glioma, the APCP treatment reduced glioma cell proliferation, while ADO treatment increased it in a similar way (Ref. 30). Azambuja et al. showed that CD73 downregulation by small interfering RNA (siRNA) or APCP treatment decreased glioma cell migration, invasion and proliferation *in vitro*, as well as rat glioblastoma progression *in vivo* (Refs 31, 32). In agreement, the therapy using an anti-CD73 monoclonal antibody in mice can initiate the adaptive antitumour immunity, leading to inhibition of breast tumour growth and metastasis (Ref. 33). Similar results were observed in a melanoma model (Ref. 34). Besides, the non-enzymatic role of CD73 has been recently explored (Refs 35, 36), highlighting the adhesive-related properties of this protein. The protein structure can control cell interaction with extracellular matrix (ECM) components, such as laminin and fibronectin. Therefore, it can mediate cancer invasion, migration and metastasis processes (Refs 6, 36, 37).

In contradiction to evidence showing the inductive impact of CD73 in tumour development and progression, some studies have unveiled the role of the CD73/ADO pathway in inhibiting tumour development in a context-dependent way (Ref. 38). Studies have shown that ADO, through its receptors, can promote tumour cell death (Refs 39, 40), reduce tumour cell proliferation (Refs 41–45), inhibit cell invasion and migration (Refs 46–48) and enhance chemotherapy sensitivity (Ref. 49) in different tumour types. Cappellari et al. demonstrated in an experimental xenograft model of medulloblastoma that implanted cells overexpressing CD73 formed smaller tumours with reduced vascularization and enhanced apoptosis rates when compared to controls (Ref. 50). Similar results were observed using agonists of adenosine receptors in animal models of melanoma (Refs 51, 52), colon (Refs 53, 54), prostate (Ref. 55) and hepatocellular carcinoma (Ref. 56). The A3 receptor agonist has also been used in a clinical trial to treat hepatocellular carcinoma, showing to be safe and well tolerated by patients (Ref. 57).

Bowser et al. showed that CD73 is markedly downregulated in poorly differentiated and advanced-stage endometrial human carcinoma compared to normal endometrium and low-grade tumours. Using animal model, they found that ADO, via A1 receptor, promotes epithelial integrity protection by promoting cortical actin polymerization, being essential to preventing migration, invasion and metastasis (Ref. 58). Downregulation of CD73 in ovarian carcinoma and advanced breast cancer has also been reported (Refs 59, 60).

Therefore, it can be observed that adenosinergic signalling in the tumour process is dependent on the target tissue and specific characteristics of tumour cells. In this context, the role of CD73/ADO in cancer is not clear. The results observed in different experimental models are complex puzzles that involve many factors to be still understood.

## Cervical cancer

Cervical cancer is one of the most common diseases in women around the world, being the fourth main cause of cancer death in women (Refs 61, 62). Histologically, there are different types of cervical carcinoma. The most common type is squamous cell carcinoma (SCC), followed by adenocarcinoma (AC) (Ref. 63). Several histological and cytological nomenclature systems have been developed (Ref. 64). In the late 1980s, the Bethesda Classification of the cervical cytology categorized the precursor lesions in the uterine cervix in low-grade (LSIL) or high-grade squamous intraepithelial lesions (HSIL) (Ref. 65). The cervical histology grading was classified by cervical intraepithelial neoplasia (CIN) system, comprising low-grade lesions (CIN1) and high-grade lesions (CIN2/3) (Refs 15, 66).

HPV infection is caused by a DNA non-enveloped virus from the Papillomaviridae family (Ref. 67). HPV is a group of more than 200 related viruses, but approximately 54 of them are able to infect the reproductive tract mucosa and 12 of them are oncogenic types classified as high-risk HPV (Ref. 68). The HPV infection, in particular, the oncogenic types HPV 16 and 18, is detected in the vast majority of cervical cancers (approximately 99% of cervical tumours), being the most important cause of this tumour type (Ref. 69). Most HPV infections clear up on their own without treatment, but some of them progress to HSIL and invasive cervical cancer (<1% of HPV infections) (Refs 70, 71).

Two oncoproteins known as E6 and E7 are encoded by HPVs, and they are directly responsible for the development of HPV-induced carcinogenesis (Ref. 72). The activity of E6 and E7 viral genes is present in high- and low-risk types of HPV, but only in high-risk types, their action is sufficient to trigger preneoplastic lesions and cancer, deregulating normal cell cycle, promoting telomerase activity, disrupting immune cells response and providing cellular factors necessary for productive viral replication (Refs 73, 74).

In face of the complexity of cervical carcinogenesis, over the last years, numerous studies have focused in providing an understanding of the pathophysiologic mechanisms of this disease, underlying molecular mechanisms and cellular pathways, such as Wnt/β-catenin, PI3K/AKT, RAF/MEK/ERK and adenosinergic pathway (Refs 19, 75).

The RAF/MEK pathway is involved in the regulation of cancer proliferation, differentiation, angiogenesis and survival. RAS, which is a member of the RAF family, is activated in 20% of human cancers, including cervical cancer (Ref. 76). The activation of the RAF /MEK/ERK pathway promotes proliferation and invasion of cervical cancer cells (Ref. 77), as well as promotes malignant conversion of HPV-infected keratinocytes, contributing to cancer invasiveness (Ref. 76). Interestingly, the transformation and carcinogenesis of human keratinocytes expressing HPV also requires the activation of the Wnt pathway (Ref. 78). The Wnt signalling pathway plays critical roles in cell fate, proliferation, migration and tissue homeostasis (Ref. 75). In cervical cancer, aberrant Wnt/β-catenin pathway activation promotes proliferation and inhibits apoptosis (Ref. 19). Moreover, this pathway contributes to cervical cancer cell migration and invasion by regulating epithelial-to-mesenchymal transition (EMT) (Ref. 79). Another pathway that is often dysregulated in cervical malignancy is PI3K/AKT. This pathway is considered a biomarker for poor prognosis in cervical cancer (Ref. 80), being related with metastasis and tumour recurrence (Ref. 81). It has been shown that oncoproteins of HPV are associated with the PI3K/AKT pathway, contributing to the tumour initiation, cell proliferation, metastasis, angiogenesis and resistance to therapy (Ref. 82). The signal transduction pathways present a central role in tumour development and progression, being the focus in several studies, including preclinical and clinical approaches (Ref. 75). The influence of purinergic signalling in cervical cancer progression has also been explored since this pathway is implicated in several tumoural mechanisms, including immune response, inflammation, cell proliferation, differentiation and cell death (Ref. 83).

## Adenosinergic pathway in cervical cancer: the example of dual face of CD73/ADO

CD73 is a major enzyme producing ADO from extracellular AMP. Furthermore, the influence of CD73 in tumour growth is not only limited to its enzymatic function but also related to its non-enzymatic action (Ref. 6). The protein structure of CD73 can control cell–cell adhesion properties and cell interaction with ECM components, mediating cancer invasion, migration and metastasis (Ref. 6).

CD73/ADO plays a role in several physiological and pathological cell processes, including vasodilatation, neurotransmission, tissue homeostasis and acute and chronic inflammation (Ref. 84). More recently, CD73 has gained considerable attention as a target for cancer treatment (Ref. 85). CD73 is upregulated in the majority of human solid tumours in comparison to normal tissues. Among these tumours, we can mention glioblastoma (Ref. 85), thyroid carcinoma (Ref. 86), pancreatic carcinoma (Ref. 87), renal cell carcinoma (Ref. 88) and colorectal carcinoma (Ref. 89). However, in general, in cancers of the genitourinary system, such as endometrial, ovarian, uterine and prostate, a lower expression of CD73 is observed (Ref. 90).

In cervical tumours, the role of CD73/ADO is a bit controversial (Ref. 90). Our research group has already demonstrated that different cervical cancer cell lines express ectonucleotidase members at different levels and show different hydrolysis patterns of adenine nucleotides (Ref. 91). Whereas SiHa (HPV+) and HeLa (HPV+) cancer cell lines presented similar levels of CD73 expression and AMP hydrolysis, in C33A (HPV-) cells, the expression of this gene and its enzymatic activity were almost undetectable (Ref. 91).

Just like CD73 expression can fluctuate among cell types, the impact of the adenosinergic pathway on cervical cancer development and progression is not completely clear. Studies targeting this issue are presented below.

### CD73/ADO axis supporting cervical cancer progression

In cervical cancer, the CD73 expression has been linked to the regulation of different tumour mechanisms, such as chemoresistance, immunosuppression, cell proliferation and migration (Ref. 92).

Regarding drug resistance mechanisms, Carrera-Martínez et al. showed that knockdown of CD73 expression by siRNA or cell treatment with the A2A receptor antagonist significantly decreased multidrug resistance protein-1 (MRP1) expression and made cervical cancer cell lines more sensitive to cisplatin treatment (Ref. 92).

Besides chemoresistance, there are other pieces of evidence showing that the expression of CD73 and production of ADO can create an immunosuppressive microenvironment, facilitating tumour development and progression. Mora-García et al. showed that HPV+ cervical cancer cells expressed high levels of CD73, producing large amounts of ADO in the presence of AMP, which in turn impaired CD8+ T cells’ functionality (Ref. 93). In addition, the activation of A2B receptor by ADO in CaSki and HeLa cell lines induced an increase in the production of immunosuppressive IL-10 by cancer cells, protecting them from T cell-mediated cytotoxic lysis (Ref. 94). Likewise, the higher levels of CD39 and CD73 expressed by mesenchymal stromal cells (MSCs) derived from human cervical cancer tumours can contribute to ADO production and the mechanisms to evade antitumour response (Refs 95, 96).

The expression of CD73 is also related with proliferation and migration in cervical cancer. Gao et al. showed that CD73 overexpression induced proliferation and migration of Hela and SiHa cells. However, surprisingly, the treatment of cells with a high concentration of extracellular ADO (>100 μM) had the opposite effect, evidencing that the increase in proliferation and migration of cervical cancer cells is not always associated with CD73 enzymatic activity (Ref. 35). In a similar way, CD73 overexpression significantly promoted cervical cancer cells’ proliferation *in vitro* and tumour growth *in vivo*, via the EGFR/AKT1 pathway (Ref. 97).

### CD73/ADO axis inhibiting cervical cancer progression

In contrast to the data addressed above, there is evidence showing that, unlike the majority of solid cancer types, an inverse correlation between the adenosinergic axis and cervical tumour development and progression has been reported (Refs 3, 98–100). Curiously, the same is observed in other tumours of the genitourinary system, such as ovarian serous cystadenocarcinoma, testicular germ cell tumours, endometrial carcinoma, uterine carcinosarcoma, bladder carcinoma, kidney and prostate adenocarcinoma (Ref. 98).

Our research group investigated the expression and activity of CD73 in sphere-forming cells from cervical cancer in comparison to monolayer cells *in vitro.* It is known that low adherent tumourspheres are enriched in stem-like cancer cells (Ref. 101), which have tumour-initiating ability and play a critical role in tumour metastasis, relapse and chemoresistance (Ref. 102). Our results evidenced a reduction in CD73 expression and enzymatic activity in cervical spheres when compared to monolayers (Ref. 99). Interestingly, our *in silico* analyses have supported our *in vitro* results, showing that three-dimensional spheres derived from cervical, thyroid and breast cancer presented decreased expression of CD73, when compared to their adherent counterparts (Ref. 99).

Gao et al. showed that ADO treatment inhibited the migration and invasion of Hela and SiHa cells via repressing the EMT program. The authors showed that after treatment of tumour cells with ADO, the epithelial marker E-cadherin was significantly increased, while the mesenchymal markers N-cadherin and fibronectin were decreased. In addition, ADO induced cervical cancer cell apoptosis, as confirmed by analysing the expression levels of apoptosis-related molecules (Ref. 103). Another study reported that the treatment with ADO analogue decreases the viability of HeLa and SiHa cells through induction of necrosis and apoptosis, respectively (Ref. 104).

In accordance, Mello et al. showed that ADO uptake produced from ATP degradation by ectonucleotidases plays a crucial role in inducing apoptosis in the human cervical cancer cell line (Ref. 100). ADO uptake promoted AMPK activation, deoxy-ATP (dATP) accumulation and increased p53 level, culminating in induction of apoptosis and autophagy (Ref. 100). The authors confirmed the results by treatment of cervical tumour cells with dipyridamole (an adenosine transporter inhibitor), which induced almost complete recovery of cell viability (Ref. 100). Indeed, in the same work, the authors observed that when SiHa cells were treated with different ADO concentrations (from 0.1 mM to 5 mM), only the higher concentrations of ADO (2 mM and 5 mM) reduced cell viability (Ref. 100). In the same way, Monroy-Mora showed that high ADO levels in the TME of cervical cancer cells, caused by ADA inhibition, decreased the viability of cell lines by inducing apoptosis. When cells were treated with ADO at a concentration of 1000 μM in the presence of EHNA, a specific ADA inhibitor, the viability of C33A, CaSki and HeLa cells decreased by 60%, 83% and 96%, respectively. On the other hand, the presence of ADA conferred protection to cervical tumour cells against the cytotoxic effect of high ADO content (Ref. 105). These results reinforce the idea that nucleoside concentration may be critical to the effects produced on cervical tumour cells (Table 1).

**Table 1.** Studies using different conditions of treatment and concentrations of adenosine (ADO) in cervical cancer cells

Authors	Cell line	Adenosine treatment	Effect
De Andrade Mello P, 2014	SiHa	≥ 2 mM, 24 h	Reduction of cell viability
Gao ZW, 2016	HeLa and SiHa	≥ 100 μM, 24 h	Reduction of migration and invasion
		500 μM, 48 h	Induction of apoptosis
		-	Inhibition of EMT
Gao ZW, 2017	HeLa and SiHa	≥ 500 μM (HeLa); ≥ 1 mM (SiHa), 24 h and 48 h	Reduction of cell viability
		≥ 100 μM, 24 h	Reduction of migration
García-Rocha R, 2019	CaSki	1 mM (24 h, 48 h, 72 h, 96 h)	Increase in production and expression of TGF-β1
	HeLa	1 μM (96 h), 10 μM, 100 μM, 1 mM (72 h and 96 h)	Increase in production and expression of TGF-β1
Monroy-Mora, A, 2022	C33A, CaSki and HeLa	1000 μM, 48 h	Inhibition of cell viability by 6% (C33A), 10% (CaSki) and 20% (HeLa)
Mora-García ML, 2017	CaSki, SiHa, HeLa and RoVa	500 μM, 5 h	Inhibition of (>50%) proliferation, activation and cytotoxic activity of CD8+ T cells
Sinha S, 2007	HeLa and SiHa	Adenosine analogues: 200 μg/mL, 24 h	Necrosis in HeLa cells and membrane blebbing and formation of apoptotic bodies in SiHa

## Analysis of modulation of CD73 and other adenosinergic pathway components in cervical cancer

In spite of the considerable number of studies linking the roles of CD73/ADO to the development process or prognosis of cervical cancer, as presented in this review, the results obtained by different authors are even contradictory. In this context, comprehensive bioinformatics methods have made possible to find an association among other genes and cellular pathways. Given that, to clarify how the expression of CD73 is in cervical cancer and how this enzyme is related to cancer progression, we conducted bioinformatics analysis using various transcriptomic databases. Additional materials and methods information is provided in Supplementary Material.

### Expression of CD73 in cervical cancer cell lines

First of all, we decided to investigate the expression of CD73 in different cervical cancer cell lines from the GSE9750 dataset, obtained from the Gene Expression Omnibus (GEO) database. In Figure 2, it is possible to observe that the expression of CD73 diverges widely among the different cells analysed. The C-33A lineage showed lower expression of CD73, while SW756 presented higher expression levels. SiHa and HeLa cells, which are the most common cell lines used in studies about cervical cancer, presented a very similar level of CD73 expression. In accordance, the enzymatic activity for AMP hydrolysis evaluated on the cell surface of SiHa, HeLa and C-33A cells showed that C-33A has smaller degradation rate levels compared with SiHa and HeLa that are very similar (Ref. 91). The wide range of variation in the expression levels of CD73 found in this analysis can explain, at least in part, the contrasting results observed in studies using cancer cell lines, both *in vitro* and *in vivo.*

It is important to highlight that the cell lines whose CD73 expression is lower (C-33A and HT-3) in our analysis are negative for HPV DNA or RNA. In contrast, the cells with the highest CD73 expression are positive for HPV (Refs 106–108). This observation comes together with the results obtained by Mora-García et al., which showed that cells positive for HPV-16 obtained from cervical samples of CIN1 patients presented higher CD73 expression when compared to HPV-16 negative samples (Ref. 109). The same result was observed when comparing the expression of CD73 in cervical carcinoma cell lines positive or negative for HPV (Ref. 93). All together, these findings could indicate that HPV infection is able to modulate CD73 expression in cervical cancer.

### Expression of CD73 in tumour tissue

Our next step was identifying whether there are differences in the expression of CD73 in human tissues of cervical cancer in comparison to non-tumour cervix tissue. For that, we analysed ten microarray datasets (GSE29570, GSE39001, GSE67522, GSE7410, GSE9750, GSE7803, GSE52903, GSE63514, GSE6791 and GSE27678) obtained from the GEO database and The Cancer Genome Atlas Cervical Squamous Cell Carcinoma and Endocervical Adenocarcinoma (TCGA-CESC) database (Supplementary Table 1). Our analysis revealed a significant downregulation of CD73 in samples of human cervical cancer tissue when compared to non-tumour tissue in the majority (8/11; 72.7%) of cohorts analysed (Figure 3). Among them, two datasets (GSE6791 and GSE63514) did not present differences between cancer tissue and non-tumour tissue, and one of them presented upregulation of CD73 (GSE27678) (Figure 3).

### CD73 expression × FIGO staging system for cervical cancer

The FIGO (International Federation of Gynecology and Obstetrics) staging system is used for staging classification of carcinoma of the cervix uteri. The cervical cancer stage ranges from stages I (1) through IV (4) (Ref. 65). Thus, next, we evaluated the expression of CD73 in higher FIGO stages compared to lower FIGO stages. The presence or absence of lymph node metastasis (LNM) is essential to the FIGO statement (Ref. 65) since it facilitates prognosis evaluation. Therefore, in the present study, we included this variable in our analysis. The data analysed here were obtained from GEO databases and the TCGA-CESC database. Interesting, there was no correlation between the CD73 expression and FIGO staging (Supplementary Figure 1 and Supplementary Table 2). Similarly, there was no difference comparing the expression of CD73 in samples with the presence or absence of LNM (Supplementary Figure 2 and Supplementary Table 3).

In sequence, we used the GSE7803 dataset, with samples of high-grade squamous intraepithelial lesions (HSIL) of the cervix and invasive squamous cell carcinomas (SCC) to investigate whether CD73 expression could be linked to cervical cancer progression. We observed a significant downregulation of CD73 in SCC compared with normal squamous cervical epithelium (NC) or with HSIL (Figure 4), reinforcing the idea of a negative correlation between CD73 expression and tumour development.

### CD73 expression and survival rate

In our conception, the reduced expression of CD73 in cervical cancer may be associated with cancer development or with the transition from a healthy cell to a tumour cell, passing through different degrees of lesions, without necessarily impacting the outcome of disease. Survival curves reinforce this idea indicating that CD73 expression did not significantly affect disease-free survival (HR = 1.2, *p*(HR) = 0.58) or overall survival (HR = 1.6, *p*(HR) = 0.071) in patients from the TCGA-CESC database when samples were categorized into two groups of low (*n* = 146) and high (*n* = 146) CD73 expression, based on the median level of CD73 expression (Figure 5A and B, respectively).

The multivariate Cox regression analysis results showed that clinical stage, age, histological grade, CD73 expression and methylation sites were not prognostic factors, not influencing the patient’s risk of death (Figure 5C).

To evaluate whether CD73 expression could be related to other outcome factors, we performed univariate analysis, and the results showed that CD73 expression was not associated with age ≥ 50 years old (*p* = 0.1137), keratinizing versus non-keratinizing squamous cell carcinoma (*p* = 0.1421), presence of regional lymph node (*p* = 0.856), distant metastases (*p* = 0.681) or between histological grades G1/G2 versus G3/G4 (*p* = 0.3489) (Supplementary Table 4). Although clinical stages I/II versus III/IV and tumour stages (T1, T2 and T3/T4) have not shown statistical differences (*p* = 0.1038 and *p* = 0.122, respectively), we can observe a reduction in CD73 expression associated with disease progression.

It is essential to highlight a limitation of this analysis since they were performed using only one dataset (TCGA-CESC). The TCGA cervical cohort is composed mainly of patients with FIGO stage I disease, being not comparable to patients with advanced cervical cancer. Thus, for a more accurate result, it would be necessary to evaluate samples from patients with more advanced tumour stages. In addition, as shown in Figure 5, we considered confounding factors such as clinical stage, patient age and tumour stage/grade, but not others like treatment and HPV type, which might lead to potential bias. Other datasets either have none or very few cervical cancer-related data or often lack critical clinical information about survival time. Additionally, the analyses conducted are exclusively *in silico.* While valuable for initial insights, they require further validation through experimental studies using fresh tumour samples. Future research should focus on addressing these gaps to provide a more thorough validation of the results presented.

### The modulation of other adenosinergic components in the cascade

It is essential to keep in mind that the purinergic system accounts for a complex network of enzymes and receptors responsible for the generation, recognition and degradation of extracellular ATP and ADO. However, although we are focusing our attention on CD73, other factors contribute to regulating extracellular ADO levels. For example, ADK is an intracellular enzyme able to metabolize intracellular ADO. Thereby, the modulation of ADK activity by different factors can interfere with extracellular levels of ADO. If ADK is inhibited, there is an increase in intracellular concentrations of ADO, favouring the release of ADO to extracellular space. Moreover, the downregulation of nucleoside transporters can enhance the concentrations of extracellular ADO (Ref. 3).

ADO can also not bind to its receptors to exert their effects but instead be metabolized to inosine by secreted ADA (ecto-ADA) or yet be transported into cells by equilibrative and concentrative transporters (ENTs and CNTs), which can also mediate nucleoside efflux (Ref. 3). All of these alternatives, taken together, can regulate ADO metabolism. Therefore, we decided to analyse other components that could regulate ADO concentrations.

In our *in silico* analysis, we observed an imbalance of adenosinergic pathway components (Figure 6). Among nine datasets analysed from GEO, in six of them (66.7%) (GSE67522, GSE52903, GSE39001, GSE29570, GSE9750 and GSE63514), the tumour samples presented enhanced expression of at least one member of CNT (*SLC28A1*, *A2* and *A3* genes) and ENT protein families (*SLC29A1*, *A2*, *A3* and *A4* genes) when compared to normal tissue samples (Figure 6). These transporters can transfer ADO into cells, reducing the levels of this nucleoside in extracellular space. Additionally, all datasets analysed presented a reduction of the surface glycoprotein dipeptidyl peptidase 4 (*DPP4/*CD26) expression, and eight of them (88.9%) presented enhanced ADA expression (Figure 6).

ADA catabolizes ADO to inosine, thus downregulating the biologic effects of extracellular ADO. However, ADA does not have its own transmembrane domain and is associated with CD26. CD26 works as a binding protein for extracellular ADA, anchoring it to the cell membrane (Ref. 110). Interestingly, CD26 also binds ECM proteins such as fibronectin and collagen. Consequently, the decreased expression of CD26 has been linked to increases in tumour invasion and metastasis (Refs 111, 112). Additionally, we observed that the expression of TNAP, a membrane-bound phosphatase, is decreased in tumour samples of 55.5% of datasets analysed (Figure 6). This result reinforces that in addition to CD73, other enzymes able to hydrolyze AMP to ADO, such as TNAP, are also downregulated in cervical cancer.

### CD73 is epigenetically modulated in cervical cancer

DNA methylation is a common epigenetic signalling mechanism that cells use to modulate gene expression. Therefore, it is an important component in numerous cellular processes, including many cancer types. DNA methylation is related to suppression of gene expression by blocking the promoters (Ref. 113). However, it is also associated with gene bodies or repeated sequences and, in some cases, with gene activation or heterogeneous gene expression (Refs 113, 114). Mechanistically, the downregulation of NT5E mRNA levels was shown to be modulated by hypermethylation in the cytosine-phosphate-guanine (CpG) island located in the regulatory region of the CD73 gene in breast cancer (Ref. 115) and in both primary and metastatic melanomas (Ref. 116).

Using an artificial intelligence tool, our research group successfully demonstrated the potential of combining mRNA levels of *NT5E* with its DNA methylation levels as a differentiating factor between thyroid cancer and normal thyroid tissues (Ref. 20). Recent studies have shown genes that might be downregulated due to hypermethylation in cervical cancer samples in comparison with normal cervical samples (Refs 117, 118). Interestingly, one of these genes is the *DPP4* gene, which is downregulated in cervical cancer samples, as can be observed in our *in silico* analysis (Figure 6).

Considering these studies, we hypothesized that the CD73 downregulation in cervical samples could be a result of abnormal methylation in distinct genomic regions of the *NT5E* gene, which encodes CD73. As shown in the schematic representation of the CD73 structure in Figure 7A and Supplementary Table 5, we collected 17 probes from publicly available datasets that covered the transcription start site (TSS) in the promoter region, first exon, body, and three prime untranslated regions (3′-UTR) of CD73.

First, the methylation analysis and gene expression data from TCGA-CESC revealed a significant inverse correlation between *NT5E* mRNA expression and methylation levels of 10 out of 15 potential methylation sites (*p* < 0.0001, *n* = 306; Figure 7B and Supplementary Table 5), with most strong correlation within the promoter region ((2) cg27039625: ρ = −0.453; (4) cg13315970: ρ = −0.544; (5) cg21730993: ρ = −0.532; (6) cg10663055: ρ = −0.529) and another one site in the CD73 (*NT5E*) body ((11) cg27297263: ρ = −0.569).

Next, we compared the CpG methylation status of normal and cervical cancer in methylation array in the TCGA-CESC, GSE20080, GSE30760, GSE36637, GSE99511, GSE37020, GSE41384, GSE134772 and GSE46306 datasets separately considering its β-values processed. Our results from TCGA-CESC, GSE46306 and GSE134772 datasets indicate that CpG sites within on the *NT5E* promoter were significantly hypermethylated in cervical cancer samples in comparison to standard cervical samples (adjusted *p*-value < 0.05) (Figure 8). There is no significant difference between cancer and standard cervical samples in the GSE99511 dataset. The probes targeting the CpG sites with the *NT5E* promoter were not covered on the HumanMethylation27K array used for analyses of the GSE20080, GSE30760, GSE36637, GSE37020 and GSE41384 datasets.

cg10663055, cg21730993, cg27039625, cg13315970 and cg27297263 CpGs showed a significant relation with CD73 expression (*p* ≤ 0.05 or Pearson’s ≥ 0.4). We used these sites to generate a linear regression model for predicting the impact of methylation on the levels of CD73 expression. As a result, the coefficient of correlation (*R*) was 0.558, and the adjusted *R*2 was 0.312, indicating that about 31.2% of the decreased expression of CD73 could be explained by the hypermethylation of CD73 in these sets of five CpGs.

ADK controls the clearance of intracellular ADO by its conversion to AMP, using ATP as a phosphate donor. Studies have shown that a higher expression of ADK-L and, consequently, low levels of ADO lead to an increase in global DNA methylation (Ref. 60). Conversely, low levels of ADK-L and consequently high levels of ADO prevent DNA methylation and lead to global DNA hypomethylation. Thus, we analysed if there is a relationship between levels of *NT5E* and ADK from the TCGA-CESC cohort. As a result, the levels of *NT5E* exhibit a significant and weak negative correlation with the levels of ADK (*r* = −0.1364; *p* = 0.0166; *n* = 308) (Supplementary Figure 3A). We also noticed that ADK expression was higher in cervical cancer than in normal cervix samples (0.4845 ± 0.0369, *n* = 305 vs −1.257 ± 0.142, *n* = 3; *p* < 0.0001) (Supplementary Figure 3B). Further studies could better investigate this question considering the expression of both isoforms of ADK, nuclear (ADK-L; long isoform) and cytoplasmic (ADK-S; short isoform), since ADK-S is involved mainly in the purine salvage pathway, while ADK-L drives the intranuclear methylation reactions (Ref. 18).

In other cancer types, such as breast cancer and melanoma, the relation between higher gene methylation and downregulation of the *NT5E* gene has already been shown (Refs 21, 116). In breast cancer, the methylation level of the *NT5E* gene was significantly higher when compared with normal breast tissues and associated with poor prognostic factors, such as large tumour size, high histologic grade, negative oestrogen receptor expression and negative Bcl-2 expression (Ref. 21). In the same way, the *NT5E* gene was downregulated by methylation in melanoma cell lines and patient melanoma samples (Ref. 116).

## Clinical applications

Cervical cancer is preventable through regular screening strategies, such as Pap test and colposcopy (Ref. 119). These tests in combination can detect very early stages of disease. In addition, prophylactic HPV vaccinations raise as the best choice to prevent cervical cancer (Ref. 119). Although the use of these screening and prophylactic strategies has decreased the incidence of cervical cancer, significant occurrences of this cancer still remain in certain groups, especially Hispanic/Latina and African American women (Ref. 119). Interestingly, Hispanic/Latina women have the highest incidence of cervical cancer, with the worst outcome and higher mortality rate compared to other populations (Ref. 119). Thus, the development of new treatment strategies for these patients is imperative to improve cervical cancer survival rates.

In the early stages of cervical cancer, surgery and/or radiotherapy are the treatment choice. In most aggressive cases, the standard of care involves chemotherapy and radiotherapy in combination. Unfortunately, disease recurrence can reach 30% in the early stages and increase up to 70% in the locally advanced disease (Ref. 120). Luckily, in recent years, new therapeutic strategies have been studied, including antiangiogenic agents, immunomodulatory vaccination and immunotherapy with monoclonal antibodies (Ref. 120). Several clinical trials have already been completed or are ongoing to evaluate with the use of anti-CD73 monoclonal antibody (mAb) for different types of tumours and cancers, such as MEDI9447 (oleclumab), AB680 (quemliclustat), TJ004309 (uliledlimab), JAB-BX102, PT199, AK119, BMS-986179, IBI325 and IPH5301 (for details of clinical trials, see Supplementary Table 6). However, only two of them are available to cervical cancer patients (NCT03454451: CPI-006 (mupadolimab) and NCT04672434: Sym024 – https://www.clinicaltrials.gov Accessed on 08/03/2024).

A clinical study involving cervical cancer patients treated with mupadolimab has been concluded. Although the study does not specifically mention patients with cervical cancer, it included 34 patients with various types of advanced cancers, who received intravenous doses ranging from 1 to 24 mg/kg every 21 days. Immunological analysis of patient blood samples revealed a slight decline in circulating CD73-positive B cells immediately after infusion, with levels returning to baseline by day 21. However, the researchers observed that this reduction was not due to blockade by the administered antibody since reactivity with another non-blocking anti-CD73 antibody, AD2, was similarly reduced. Interestingly, patients with non-small cell lung, head and neck and prostate cancers exhibited the most significant changes in circulating B cells and were more likely to experience tumour volume reduction. This observation suggests that mupadolimab may serve as an effective immunotherapy for cancer, owing to its capacity to activate B cells, generate memory B cells and stimulate the production of antigen-specific antibodies. Moreover, the treatment was generally well tolerated, with only a few patients experiencing grade 3 adverse events. This phase 1 trial is now evaluating mupadolimab in combination with adenosine A2A receptor blockade, in combination with pembrolizumab, and in a triplet regimen (Ref. 121).

Although the clinical study with Sym024 is not yet complete, the preliminary results presented at the 2024 Cancer Research Annual Meeting are promising. The authors reported minimal adverse effects among the 43 patients treated as from October 12, 2023. Target engagement assessed both peripherally (free soluble CD73) and within tumours (enzyme activity), demonstrating a sustained reduction in free soluble CD73 in the blood at dose levels of ≥1500 mg. Additionally, dose-dependent CD73 modulation was observed, with over 80% inhibition achieved in tumour biopsies at doses of ≥1500 mg, suggesting that Sym024 may effectively prevent adenosine-mediated tumour evasion and support further clinical investigation (Ref. 122).

However, further efforts in basic and translational research are still needed to reach significant advances in anti-CD73 mAb-based immunotherapies, particularly for cervical cancer. This is important because clinical studies with oleclumab, one of the most extensively evaluated anti-CD73 mAbs to date, have yielded conflicting results. In a phase I study, oleclumab demonstrated a manageable safety profile, with minimal treatment-related adverse events. Moreover, antitumour activity was observed in cancer types that are generally resistant to immunotherapy (Ref. 123). On the other hand, in a Japanese study with advanced solid tumours, although the treatment with oleclumab was well tolerated, no disease control was achieved after 8 weeks and all six patients developed progressive disease (Ref. 124). Subsequent studies, such as the COAST and SYNERGY trials, indicated that the combination of oleclumab with durvalumab may enhance objective response rates and prolong progression-free survival compared to durvalumab alone, though without a significant increase in clinical benefit rate in certain scenarios, such as in triple-negative breast cancer (Refs 125–127). Additionally, the Neo-CheckRay trial suggested the safety of combining oleclumab with stereotactic body radiation therapy and chemotherapy, warranting further investigation in future studies (Ref. 128).

## Discussion

This review highlights the role that adenosinergic signalling plays in the plasticity of cervical cancer, which is crucial to tumour course. CD73 has been recognized to play an important role as an adhesion molecule in cancer, being able to perform different functions on tumour progression. In addition, ADO released by CD73 activity can bind to different P1 receptors, resulting in a wide range of effects. Certainly, the balance between extracellular and intracellular ADO concentrations, as well as the activity of CD39 and CD73, is an essential factor to determine what role the adenosinergic pathway will play in cancer, whether a hero or a villain.

Overall, the bioinformatics analysis performed in this study highlights the hypothesis that CD73 is inversely correlated to cervical cancer development. Our study is a pioneer in demonstrating that the downregulation of *NT5E* gene expression in cervical cancer samples in comparison to normal samples can be explained by cumulative methylation. This idea is reinforced by our *in silico* analysis showing an inverse correlation between ADK and CD73 expression. This result is significant since high levels of ADK-L induce increased methylation (Ref. 29) and has been associated with tumourigenesis, mitogenesis and invasion (Ref. 62). Meanwhile, our bioinformatics analysis showed that lower levels of CD73 did not seem to affect patients’ survival. This finding is very intriguing and opens up a field of possibilities for further studies investigating how the reduction of CD73 expression can affect tumour development.

In our conception, in some circumstances, it can be advantageous for cancer development to have reduced CD73 levels mainly because ADO has been identified as an important factor in cell death (Refs 63, 64). The uptake of extracellular ADO or intracellular ADO can induce apoptosis in cancer cells (Refs 63, 65). In cervical cancer, the high availability of ADO cytoplasmatic/extracellular leads to its conversion to AMP by ADK, culminating in AMPK activation, p53 activation and autophagy induction (Ref. 44).

Therefore, following the rationale, it seems logical that maintaining low levels of CD73 expression would culminate in reduced levels of extracellular ADO and, consequently, lower levels of intracellular ADO providing an advantage in terms of survival and proliferation to tumour cells. In addition, the reduced expression of CD73 is also accompanied by the downregulation of CD26. The lowest expression of these two molecules can be associated with the enhanced migratory and invasive ability of tumour cells since both proteins can regulate CAMs and ECM (an overview of this can be seen in Figure 9).

Unlike most other tumours that have high CD73 expression, cervical cancer seems to have low expression of this enzyme. Despite the advantages of upregulating CD73 for cancer progression in the majority of cancer types, why do some tumours, including cervical cancer, downregulate CD73? This answer remains largely unclear. In fact, the low expression of CD73 in cervical cancer is one of the most intriguing issues to discuss here (Refs 98, 129). There is evidence that it can be attributed to several potential factors related to tumour biology and the TME, as addressed below:

1. Tumour suppressor role: CD73 has been implicated as having a tumour-suppressive role in certain contexts (Ref. 58). Consequently, its downregulation might be a mechanism to promote tumour progression. For example, in endometrial carcinoma, the downregulation of CD73 makes TGF-β1 shift from being a tumour suppressor to being a promoter, impairing epithelial integrity and allowing tumour cells to develop stress fibres and macromolecule permeability, further inducing cell migration and invasion (Ref. 130).
2. Immune regulation: In the context of cancer, CD73-produced ADO typically helps tumours evade the immune system by creating an immunosuppressive microenvironment (Ref. 131). However, in some cases, cancer cells might downregulate CD73 as part of a complex mechanism to adapt to specific microenvironmental conditions. For example, in ovarian carcinoma, patients with positive CD73 expression showed better prognosis compared to the CD73 negative group. Interestingly, significantly more infiltration of regulatory T cells was observed in the CD73 negative group compared to the CD73 positive group, indicating higher immunosuppressive activity in CD73 negative tumours (Ref. 60).
3. Tumour stage: CD73 can be regulated differently in early and advanced stages of cancer. In melanoma, the activation of any ADO receptor is able to inhibit tumour growth only at its early stage, but at a more advanced tumour stage, the stimulation of ADO receptors is related to enhanced tumour cell proliferation (Ref. 42).
4. Epigenetic regulation: Although little explored, recent studies have pointed to abnormal patterns of *NT5E* gene methylation during cancer progression in different tumour types, such as breast (Ref. 21), thyroid (Ref. 20), melanoma (Ref. 116), head and neck carcinoma (Ref. 132) and pancreatic carcinoma (Ref. 133). Although it has already been shown in the literature that gene expression in cervical cancer cells can be regulated by DNA methylation (Ref. 134), for the first time, here we show evidence that the *NT5E* gene can also be epigenetically regulated in cervical cancer.
5. Regulatory pathways: The adenosinergic pathway can affect tumour progression by modulating other signalling pathways. In melanoma cells, it was demonstrated that ADO induces cell survival via A3 receptor activation and it kills the cell through A2A receptor. The signalling pathway triggered by A2A receptor involves protein kinase C (PKC) and extracellular signal-regulated kinases-1 and -2 (ERK-1 and ERK-2) (Ref. 39). In addition, the treatment of melanoma cells with adenosine receptor (AR) agonist promotes antitumour activity by the Wnt pathway via its key elements GSK-3β and β-catenin (Ref. 52). In prostate cancer cells, the activation of the A3A receptor inhibits *in vivo* tumour growth and metastasis in mice and inhibits cell proliferation and invasion *in vitro.* This antitumour action involves suppression of ERK1/2, leading to reduced nicotinamide adenine dinucleotide phosphate (NADPH) oxidase activity by inhibiting the cyclic AMP/PKA pathway (Ref. 47). In ovarian cancer cells, the treatment of cells with A2B receptor agonists may induce apoptosis via the mitochondrial signalling pathway by activation of caspase-3, downregulation of the regulatory protein Bcl-2 and upregulation of Bax protein (Ref. 45). In cancer stem cells (CSCs) from GBM, the stimulation of ADO receptors by agonists modulates the expression of pro-apoptotic proteins significantly increasing the Bax mRNA levels and Bax transcriptional activity, thus culminating in cell apoptosis (Ref. 43).
6. Cell-type specification: The expression of CD73 can vary significantly between different cell types and tissue origins. Genitourinary cancers might inherently have different regulatory mechanisms affecting CD73 expression compared to other cancers, possibly because reproductive and urinary systems share the intermediate mesoderm as common embryological origin (Ref. 98).

Despite the above, in the current context, important questions remain unanswered: Do CD73 expression levels change during tumour progression? Could the lower expression of CD73 be important at the beginning of the tumoural process but indifferent to the progress of the disease? Could low levels of CD73 help detach some cells from the tumour mass and provide invasion in other sites? Although more studies are needed, we believe that in normal cervical tissue, CD73 is expressed in higher levels to protect epithelial integrity. In contrast, the reduction of CD73 expression in tumour cells allows tumour development and progression. What can this mean in terms of treatment? This question should be a priority when thinking about new treatment options or therapies for this tumour type.

Taken together, all this knowledge opens a window of opportunity to better understand the roles of purinergic signalling in cervical cancer and to design potential therapeutic approaches to control this disease.

## Supporting information

Iser et al. supplementary material Iser et al. supplementary material

## Supplementary material

For supplementary material accompanying this paper visit http://doi.org/10.1017/erm.2024.30. click here to view supplementary material
