# Supplementary material for: Adenosinergic Signalling in Cervical Cancer Microenvironment
Source: Expert Rev Mol Med. 2025 Jan 7;27:e5. doi: 10.1017/erm.2024.30 (PMC11707834; doi:10.1017/erm.2024.30)
Supplement: Iser et al. supplementary material [file S1462399424000309sup001.zip › Methods_Suppl.docx]

**Materials and methods**

The mRNA expression, DNA methylation and clinical data for CeCa samples were searched in two databases: the Gene Expression Omnibus (GEO; [www.ncbi.nlm.nih.gov/geo](http://www.ncbi.nlm.nih.gov/geo)) downloaded using GEOparse Python package (Python 3.6.5: Anaconda, Inc.) and the Cancer Genome Atlas (TCGA; [www.cancergenome.nih.gov](http://www.cancergenome.nih.gov)) downloaded from the UCSC XENA Browser (http:// xena.ucsc.edu).

The overall survival analysis was performed using the GEPIA2 online tool (<http://gepia.cancer-pku.cn/index.html>) which use data from TCGA.

Correlation across the TCGA cohort between EMT score and *NT5E* expression was assessed using Pearson’s correlation. For this analysis, the samples were stratified as upregulated, intermediated or downregulated considering a cut-off of *NT5E* expression of −1.5≤ or ≥1.5.

DNA methylation profile for each region of the *NT5E* gene were analyzed using available processed the methylation M- or β-values separately in each dataset as described in Table S5. Differences between groups were assessed using Mann-Whitney U or one-way ANOVA tests and p-value was adjusted for multiple comparisons using the Benjamini/Hochberg false-discovery rate (FDR) method using GraphPad Prism (Version 7). Correlations across the TCGA cohort between DNA methylation and *NT5E* expression were assessed using Spearman's rank correlation coefficient.

For differential expression analysis of *NT5E* expression, the Shapiro–Wilk test was applied to determine sample normality, multiple groups were compared using a one-way ANOVA and Tukey post-hoc test and Mann-Whitney, Student’s t-test or paired Student’s t-test was used, as appropriate using SPSS software (Version 21). Graphs were created with GraphPad Prism (Version 7).
