# Supplementary material for: Adenosinergic Signalling in Cervical Cancer Microenvironment
Source: Expert Rev Mol Med. 2025 Jan 7;27:e5. doi: 10.1017/erm.2024.30 (PMC11707834; doi:10.1017/erm.2024.30)
Supplement: Iser et al. supplementary material [file S1462399424000309sup001.zip › Table S1.docx]

| Dataset | Sample | Mean ± SD | P value | *NT5E* expression |
| --- | --- | --- | --- | --- |
| GSE29570 | Non-tumor (n=17) | 7.715 ± 0.910 | <0.0001 | ↓ |
|  | Tumor (n=45) | 7.384 ± 0.102 |  |  |
| GSE39001 | Non-tumor (n=17) | 7.715 ± 0.912 | <0.0001 | ↓ |
|  | Tumor (n=62) | 6.221 ± 0.713 |  |  |
| GSE67522 | Non-tumor (n=22) | 235.6 ± 119.5 | 0.0162 | ↓ |
|  | Tumor (n= 20) | 117.7 ± 91.15 |  |  |
| GSE7410 | Non-tumor (n=5) | 0.502 ± 0.181 | <0.0001 | ↓ |
|  | Tumor (n=35) | -0.183 ± 0.194 |  |  |
| GSE9750 | Non-tumor (n=24) | 362.1 ± 133.2 | 0.0061 | ↓ |
|  | Tumor (n=33) | 81.82 ± 16.19 |  |  |
| GSE7803 | Non-tumor (n=10) | 8.949 ± 0.402 | 0.0051 | ↓ |
|  | Tumor (n=28) | 7.796 ± 0.182 |  |  |
| GSE52903 | Non-tumor (n=17) | 8.902 ± 0.253 | <0.0001 | ↓ |
|  | Tumor (n=55) | 7.236 ± 0.151 |  |  |
| GSE63514 | Non-tumor (n=24) | 7.928 ± 0.402 | 0.5103 | Ø |
|  | Tumor (n=28) | 7.273 ± 0.175 |  |  |
| GSE6791 | Non-tumor (n=8) | 7.391 ± 0.413 | 0.6705 | Ø |
|  | Tumor (n=20) | 7.183 ± 0.258 |  |  |
| GSE27678 | Non-tumor (n=13) | 4.778 ± 0.5277 | 0.0091 | ↑ |
|  | Tumor (n=28) | 5.830 ± 0.3672 |  |  |
| TCGA | Non-tumor (n=3) | 4.590 ± 0.2680 | 0.0031 | ↓ |
|  | Tumor (n=304) | .091 ± 0.0792 |  |  |

**Table S1.**  Analysis of CD73 expression in different datasets, comparing tumor and non-tumor tissue.

NA: No statistical analysis was applied due to the small number of non-tumor samples (n=2); TCGA: The Cancer Genome Atlas; ↓: downregulation
