# Supplementary material for: Adenosinergic Signalling in Cervical Cancer Microenvironment
Source: Expert Rev Mol Med. 2025 Jan 7;27:e5. doi: 10.1017/erm.2024.30 (PMC11707834; doi:10.1017/erm.2024.30)
Supplement: Iser et al. supplementary material [file S1462399424000309sup001.zip › Table S2.docx]

| Dataset | Stage | F (DFn, DFd) | P value | *NT5E* expression |
| --- | --- | --- | --- | --- |
| GSE26511 | IB1 (n=21) *vs* | (2, 36) = 0.4049 | 0.6701 | Ø |
|  | IB2 (n=11) |  |  |  |
|  | IIA (n=7) |  |  |  |
| GSE6791 | IB1 (n=10) *vs* | (2, 17) = 2.081 | 0.1555 | Ø |
|  | IB2 (n=6) |  |  |  |
|  | III-IV (n=4) |  |  |  |
| GSE29817 | I (n=10) *vs* | (3, 147) = 1.109 | 0.3473 | Ø |
|  | II (n=94) |  |  |  |
|  | III (n=39) |  |  |  |
|  | IV (n=8) |  |  |  |
| GSE7410 | IB1 (n=17) *vs* | (2, 32) = 0.4832 | 0.6212 | Ø |
|  | IB2 (n=7) |  |  |  |
|  | IIA (n=11) |  |  |  |
| GSE39001 | IB1 (n=18) *vs* | (2, 40) = 0.2517 | 0.7787 | Ø |
|  | IB2 (n=14) |  |  |  |
|  | II-III (n=11) |  |  |  |
| GSE52903 | IB1 (n=18) *vs* | (4, 50) = 1.107 | 0.3635 | Ø |
|  | IB2 (n=9) |  |  |  |
|  | II (n=8) |  |  |  |
|  | III (n=16) |  |  |  |
|  | IV (n=4) |  |  |  |
| GSE63514 | Low Grade (n=14) *vs* | (3, 88) = 0.2854 | 0.8358 | Ø |
|  | Medium Grade (n=22) |  |  |  |
|  | High Grade (n=28) |  |  |  |
|  | Squamous (n=28) |  |  |  |
| TCGA | I (n=109) *vs* | (2, 172) = 0.5518 | 0.5769 | Ø |
|  | II (n=32) |  |  |  |
|  | III (n=34) |  |  |  |

**Table S2.**  Analysis of CD73 expression in different datasets, comparing FIGO stage.

TCGA: The Cancer Genome Atlas; Ø: No statistical difference.
