# Supplementary material for: Adenosinergic Signalling in Cervical Cancer Microenvironment
Source: Expert Rev Mol Med. 2025 Jan 7;27:e5. doi: 10.1017/erm.2024.30 (PMC11707834; doi:10.1017/erm.2024.30)
Supplement: Iser et al. supplementary material [file S1462399424000309sup001.zip › Table S3.docx]

**Table S3.** CD73 expression in cervical cancer samples with presence or absence of lymph node metastasis (LNM)

| Dataset | LNM status | Mean ± SD | P value | *NT5E* expression |
| --- | --- | --- | --- | --- |
| GSE26511 | Absence (n=20) | 6.023 ± 0.154 | 0.1807 | Ø |
|  | Presence (n=19) | 5.699 ± 0.180 |  |  |
| GSE6791 | Absence (n=15) | 6.949 ± 0.220 | 0.0763 | Ø |
|  | Presence (n=3) | 8.129 ± 0.948 |  |  |
| GSE7410 | Absence (n=19) | -0.172 ± 0.044 | 0.7260 | Ø |
|  | Presence (n= 16) | -0.196 ± 0.049 |  |  |
| TCGA | Absence (n=61) | -1.107 ± 0.251 | 0.8279 | Ø |
|  | Presence (n=59) | -1.184 ± 0.242 |  |  |

Ø: no modulation
