# Supplementary material for: Adenosinergic Signalling in Cervical Cancer Microenvironment
Source: Expert Rev Mol Med. 2025 Jan 7;27:e5. doi: 10.1017/erm.2024.30 (PMC11707834; doi:10.1017/erm.2024.30)
Supplement: Iser et al. supplementary material [file S1462399424000309sup001.zip › Table S4.docx]

| Clinical parameters | | CD73 Levels ( Mean + SD, N) | *P* value |
| --- | --- | --- | --- |
| Age | *Age ≥50* | 2,267 ± 0,1187, n=118 | 0.1137 |
|  | *Age <50* | 2,008 ± 0,1052, n=191 |  |
| Keratinizing SCC indicator | *Non-keratinizing SCC* | 1,93 ± 0,1127, n=121 | 0.1421 |
|  | *Keratinizing SCC* | 2,26 ± 0,2199, n=55 |  |
| Clinical stage | *Stage I/II* | 2,171 ± 0,09282, n=231 | 0.1038 |
|  | *Stage III/IV* | 1,856 ± 0,157, n=66 |  |
| Lymph node stage | *N0* | 2,089 ± 0,1177, n=133 | 0.8586 |
|  | *N1* | 2,052 ± 0,1581, n=60 |  |
| Tumor stage | *T1* | 2,130 ± 0,1171, n=140 | 0.122 |
|  | *T2* | 1,867 ± 0,1520, n=71 |  |
|  | *T3/T4* | 1,724 ± 0,2040, n=30 |  |
| Distant metastasis | *M0* | 1,976 ± 0,1241, n=116 | 0.681 |
|  | *M1* | 1,796 ± 0,3897, n=10 |  |
| Histologic grade | *G1/G2* | 2,018 ± 0,1068, n=153 | 0.3489 |
|  | *G3/G4* | 2,177 ± 0,1352, n=119 |  |

**Table S4.** Relationship between clinical features and CD73 expression in samples from TCGA dataset
