# Supplementary material for: Adenosinergic Signalling in Cervical Cancer Microenvironment
Source: Expert Rev Mol Med. 2025 Jan 7;27:e5. doi: 10.1017/erm.2024.30 (PMC11707834; doi:10.1017/erm.2024.30)
Supplement: Iser et al. supplementary material [file S1462399424000309sup001.zip › Table S5.docx]

**Table S5.** CpG sites analyzed in CD73 gene of TCGA-CESC and GEO datasets.

| NT5E Region | CpG site | CpG site coordinate (Chr6;GRCh37) | Distance to gene | Island status | TCGA-CESC (n=306) | |
| --- | --- | --- | --- | --- | --- | --- |
|  |  |  |  |  | **Spearman correlation (ρ)** | **p-value** |
| TSS1500 | (1) cg25262528 | 86158972 | -329 | N_Shore | ND | |
|  | (2) cg27039625 | 86159096 | -205 | N_Shore | -0.453 | <0.0001 |
| TSS200 | (3) cg17644557 | 86159103 | -198 | N_Shore | -0.368 | <0.0001 |
|  | (4) cg13315970 | 86159197 | -104 | N_Shore | -0.544 | <0.0001 |
|  | (5) cg21730993 | 86159210 | -91 | N_Shore | -0.532 | <0.0001 |
|  | (6) cg10663055 | 86159251 | -50 | N_Shore | -0.529 | <0,0001 |
| 1stExon | (7) cg17488985 | 86159426 | 125 | N_Shore | -0.44 | <0,0001 |
|  | (8) cg24635468 | 86159682 | 381 | CpG Island | -0.404 | <0,0001 |
|  | (9) cg23157089 | 86159962 | 661 | CpG Island | -0.289 | <0.0001 |
|  | (10) cg17966619 | 86160162 | 861 | CpG Island | -0.378 | <0.0001 |
| NT5E Body | (11) cg27297263 | 86160468 | 1167 | S_Shore | -0.569 | <0.0001 |
|  | (12) cg00925339 | 86162725 | 3424 | S_Shelf | 0.259 | <0.0001 |
|  | (13) cg23172664 | 86169204 | 9903 | Out of | 0.190 | 0.7443 |
|  | (14) cg24702826 | 86171768 | 12467 | Out of | 0.173 | 0.0024 |
|  | (15) cg06516476 | 86174584 | 15283 | Out of | ND | |
|  | (16) cg03285617 | 86179345 | 20044 | Out of | ND | |
| 3'UTR | (17) cg09989847 | 86205036 | 45735 | Out of | 0.145 | 0.0112 |

ND: no detected.
