# Supplementary material for: Adenosinergic Signalling in Cervical Cancer Microenvironment
Source: Expert Rev Mol Med. 2025 Jan 7;27:e5. doi: 10.1017/erm.2024.30 (PMC11707834; doi:10.1017/erm.2024.30)
Supplement: Iser et al. supplementary material [file S1462399424000309sup001.zip › Table S6.docx]

**Table S6.** Clinical trials regarding the use of anti-CD73 monoclonal antibody (mAb) for different tumor types

| Clinical Trial ID | Intervention/  Treatment | Target Disease | Design | Objectives | Patient Populations | Country | Status |
| --- | --- | --- | --- | --- | --- | --- | --- |
| NCT02503774 | MEDI9447 (Oleclumab), MEDI4736 (Durvalumab) | Advanced solid tumors, including EGFR-mutated non-small cell lung cancer (NSCLC) | Phase 1, multicenter, open-label, dose-escalation and expansion study | Evaluate safety, tolerability, pharmacokinetics, immunogenicity, and antitumor activity | Adults with advanced solid tumors, including EGFR-mutated NSCLC with at least one measurable lesion | United States, Australia, Republic of Korea | Completed |
| NCT03381274 | MEDI9447 (Oleclumab), Osimertinib, AZD4635 | Advanced EGFR-mutated non-small cell lung cancer (NSCLC) | Phase 1b/2 study | Investigate the safety, tolerability, and antitumor activity of novel combination therapies administered in participants with advanced EGFRm NSCLC. | Adults with advanced EGFRm NSCLC | United States, Republic of Korea and Taiwan | In progress, not recruiting |
| NCT03267589 | MEDI9447 (Oleclumab), MEDI4736 (Durvalumab), Tremelilumab,  MEDI 0562 (Tavolimab) | Relapsed Ovarian Cancer | Phase 2, open-label, multicenter study | Obtain preliminary evidence of efficacy of novel agents for the management of relapsed ovarian cancer, and in part 2 efficacy of novel agents compared to the standard of care (SoC). | Adults with Relapsed Ovarian Cancer | Denmark, Finland, Norway | Completed |
| NCT03611556 | MEDI9447 (Oleclumab), MEDI4736 (Durvalumab) | Metastatic pancreatic cancer | Phase 1/2, dose-escalation study | Evaluate safety, tolerability, and antitumor activity of MEDI9447 with.without Durvalumab in Combination With Chemotherapy | Adults with Metastatic Pancreatic Ductal Adenocarcinoma | United States, Australia, Norway, Spain | Completed |
| NCT03616886 | MEDI9447 (Oleclumab), MEDI4736 (Durvalumab) | Triple Negative Breast Cancer (TNBC) | Phase I/II Study | Evaluate the efficacy and safety of the combination of chemotherapy (paclitaxel + carboplatin) with immunotherapy (durvalumab [anti-PD-L1] +/- MEDI9447 [anti-CD73]) in previously untreated locally recurrent inoperable or metastatic TNBC. | Female adults with recurrent inoperable or metastatic TNBC. | Belgium, France | In progress, not recruiting |
| NCT04668300 | MEDI9447 (Oleclumab), MEDI4736 (Durvalumab) | Recurrent/Refractory  /metastatic sarcoma subtypes | Phase 2 Multi-Arm Study | Investigate efficacy in treating recurrent/ Refractory /metastatic sarcoma | Adults with Multiple Sarcoma Subtypes | United States | In progress |
| NCT06060405 | MEDI9447 (Oleclumab), MEDI4736 (Durvalumab) | Resectable pancreatic ductal adenocarcinoma | Phase 2 study | Evaluate the immune activity of durvalumab and oleclumab | Adults with Resectable pancreatic ductal adenocarcinoma | Canada | In progress |
| NCT03801902 | Oleclumab (MEDI9447), Monalizumab (IPH2201), Durvalumab (MEDI4736) | Locally Advanced Non-small Cell Lung Cancer (NSCLC) | Phase 1 study | Evaluate safety of adding durvalumab / Monalizumab / Oleclumab to accelerated hypofractionated radiation therapy (ACRT) | Adults with Advanced Non-small Cell Lung Cancer (NSCLC) | United States | In progress |
| NCT04940286 | Oleclumab (MEDI9447), Durvalumab (MEDI4736), Gemcitabine,  Nab-paclitaxel | Resectable/Borderline Resectable Pancreatic Cancer | Phase 2, open-label study | Evaluate combination therapy before surgery for pancreatic cancer | Adults with resectable/borderline resectable pancreatic cancer | United States | In progress |
| NCT04104672 | AB680 (Quemliclustat), Zimberelimab (AB122), nab-paclitaxel, gemcitabine | Advanced pancreatic cancer | Phase 1, open-label, dose-escalation, and dose-expansion study | evaluate the safety, tolerability, pharmacokinetic, pharmacodynamic and clinical activity | Adults with Advanced pancreatic cancer | United States | In progress |
| NCT05001347 | TJ004309 (Uliledlimab), Atezolizumab | Advanced or Metastatic Ovarian Cancers and Selected Advanced Solid Tumors (Head and Neck Cancer, Non Small Cell Lung Cancer, Gastrointestinal Cancer, Triple Negative Breast Cancer) | Phase 2, open-label, multicenter study | Evaluate safety, tolerability, pharmacokinetics, immunogenicity, and antitumor activity | Adults with Advanced or Metastatic Ovarian Cancers and Selected Advanced Solid Tumors | United States | Completed |
| NCT04322006 | TJ004309 (Uliledlimab), Toripalimab | Advanced Solid Tumor | Phase 1/2 study | Evaluate the Safety, Tolerance, Pharmacokinetics, Pharmacodynamics and Curative Effect of Dose Escalation and Extension | Adults with Advanced Solid Tumor | China | In progress, not recruiting |
| NCT03835949 | TJ004309 (Uliledlimab),  Atezolizumab | Advanced/metastatic cancer | Phase 1, multicenter, open label open-label study | Evaluate safety and clinical activity | Adults with advanced/metastatic cancer | United States | In progress |
| NCT05174585 | JAB-BX102, pembrolizumab | Advanced solid tumors | Phase 1/2a, Multi-Center, Open-Label Study | Assess safety, tolerability, and preliminary antitumor activity | Adults with advanced solid tumors | China | In progress |
| NCT05431270 | PT199,  PD-1 inhibitor | Advanced Solid Tumors (Non Small Cell Lung Cancer,  Pancreatic Ductal Adenocarcinoma) | Phase 1, open-label, dose-escalation/expansion study | Evaluate safety, pharmacokinetics, pharmacodynamics and preliminary efficacy | Adults with Advanced Solid Tumors. | United States | In progress |
| NCT04572152 | AK119,  AK104 | Advanced or Metastatic Solid Tumors | Phase 1a/1b, Multicenter, Open-Label, Dose-Escalation and Dose-Expansion Study | Evaluate the Safety, Pharmacokinetics, and Anti-tumor Activity of AK119 (Anti-CD73) | Adults with Advanced or Metastatic Solid Tumors | Australia | In progress, not recruiting |
| NCT05559541 | AK119,  AK104 | Advanced Solid Tumors | Phase Ib/II, open-label, multicenter study | Evaluate the Safety, Tolerability, Pharmacokinetics, and Anti-tumor Activity | Adults with Advanced Solid Tumors | China | In progress |
| NCT05689853 | AK119,  AK112 | Advanced Solid Tumors | Phase 1b/2, open-label study | Evaluate the Safety, Tolerability and Antitumor Activity | Adults with Advanced Solid Tumors | China | In progress |
| NCT05119998 | IBI325  Sintilimab | Advanced solid tumors | Phase I, Open-label, Multicenter, Dose-escalation Study | Evaluate the Safety, Tolerability, and Potential Efficacy of IBI325, | Adults with Advanced Solid Tumors | China | Completed |
| NCT02754141 | BMS-986179, Nivolumab, rHuPH20 | Advanced Solid Tumors | Phase 1/2, open-label study | Assess safety, tolerability and tumor-shrinking ability | Adults with  Advanced Solid Tumors | United States | Completed |
| NCT05143970 | IPH5301;  Trastuzumab  Chemotherapy | Metastatic Breast Cancer;  Metastatic Pancreatic Cancer;  Metastatic Gastric Cancer;  Metastatic Lung Cancer;  Metastatic Ovary Cancer;  Oesophageal Cancer;  Endometrial Cancer;  Advanced Solid Tumor | Phase I, multicenter study | Assess safety and tolerability | Adults with Metastatic and/or Advanced Solid Tumors | France | In progress |
| NCT03454451 | CPI-006 (Mupadolimab), Ciforadenant, Pembrolizumab | Select advanced cancers (Non-Small Cell Lung Cancer,  Renal Cell Cancer,  Colorectal Cancer,  Triple Negative Breast Cancer, Cervical Cancer, Ovarian Cancer, Pancreatic Cancer, Endometrial Cancer, Sarcoma  Squamous Cell, Carcinoma of the Head and Neck,  Bladder Cancer,  Metastatic Castration Resistant Prostate Cancer, Non-hodgkin Lymphoma) | Phase 1/1b, open-label, dose escalation/expansion study | Evaluate CPI-006 alone or in combination with other agents | Adults with select advanced cancers | United States, Australia | Completed |
| NCT04672434 | Sym024,  Sym021 | Squamous cell carcinoma of the head and neck;  Non-small-cell lung carcinoma-adenocarcinoma;  Pancreatic ductal adenocarcinoma;  Cholangiocarcinoma;  Colorectal carcinoma;  Gastric carcinoma;  Esophageal carcinoma;  Mesothelioma;  Cervical carcinoma | Phase 1, Open-Label, Multicenter study | Assess the safety, tolerability to establish the maximum tolerated dose (MTD) and preliminary antineoplastic activity of Sym024. | Adults locally advanced or metastatic solid tumor malignancy | United States,  Canada | In progress, |
