# Supplementary figures and images for: Adenosinergic Signalling in Cervical Cancer Microenvironment
Source: Expert Rev Mol Med. 2025 Jan 7;27:e5. doi: 10.1017/erm.2024.30 (PMC11707834; doi:10.1017/erm.2024.30)

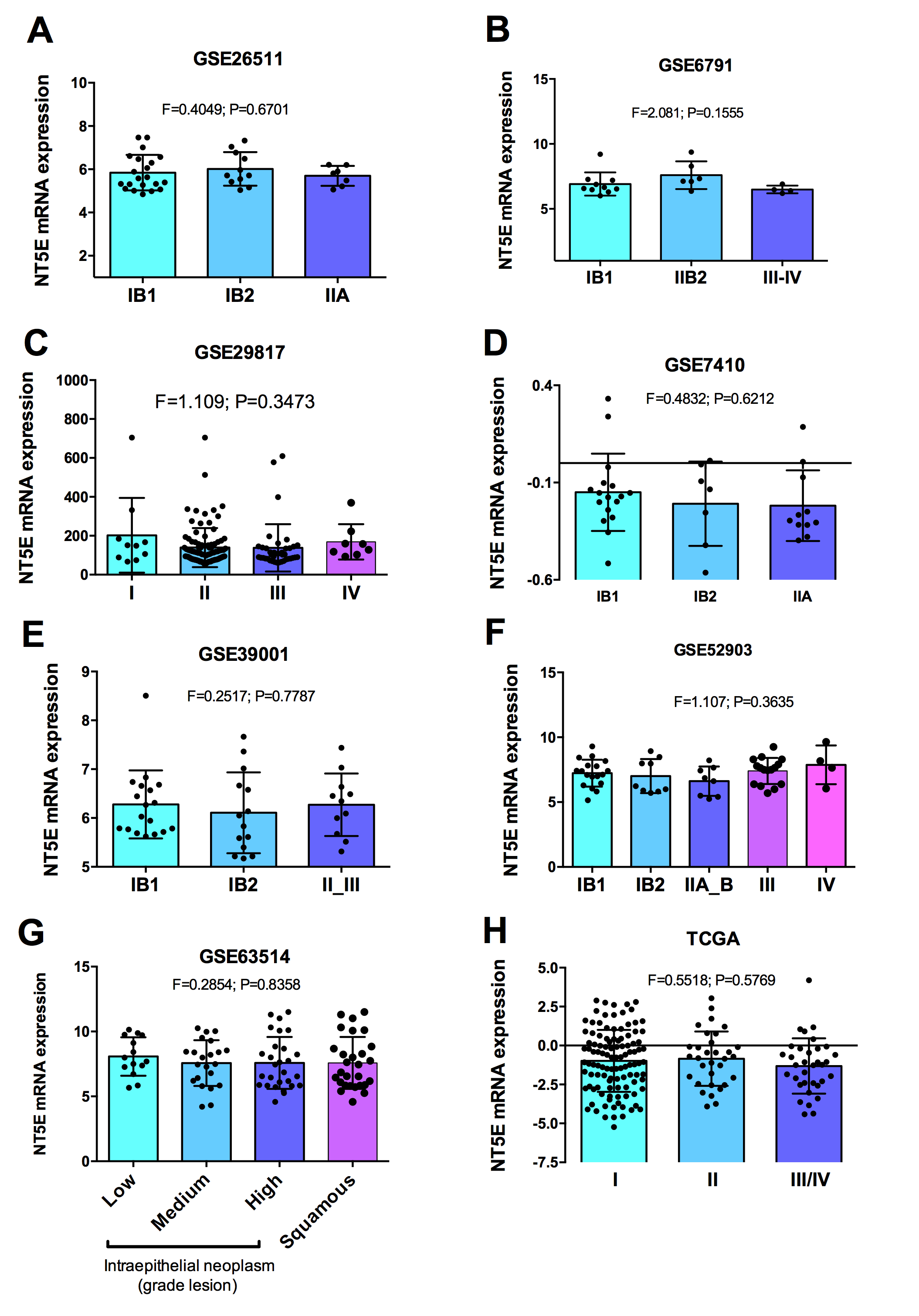

Supplement: Iser et al. supplementary material [file S1462399424000309sup001.zip › Figure S1_FIGO.tiff]

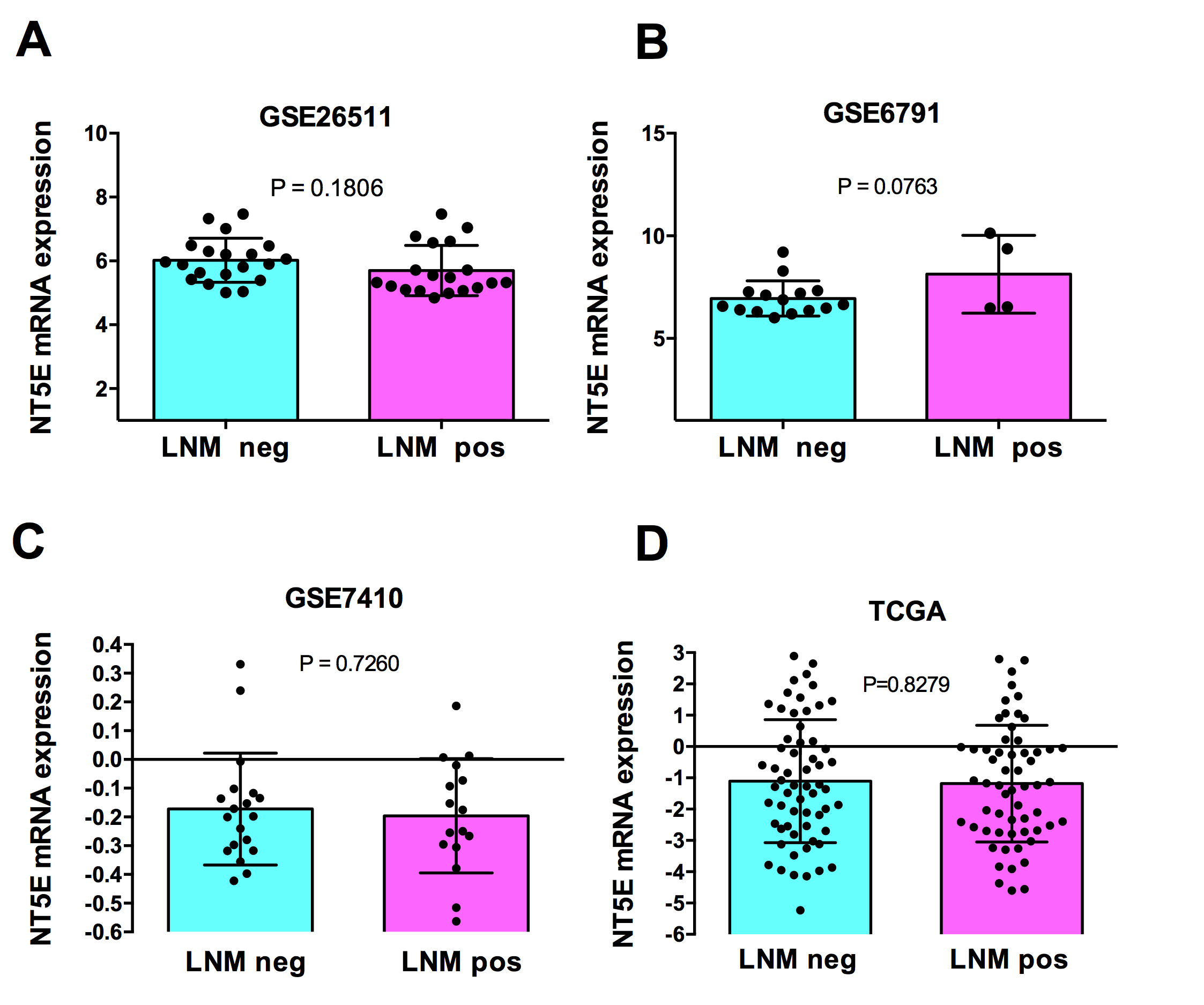

Supplement: Iser et al. supplementary material [file S1462399424000309sup001.zip › Figure S2_LNM.tiff]

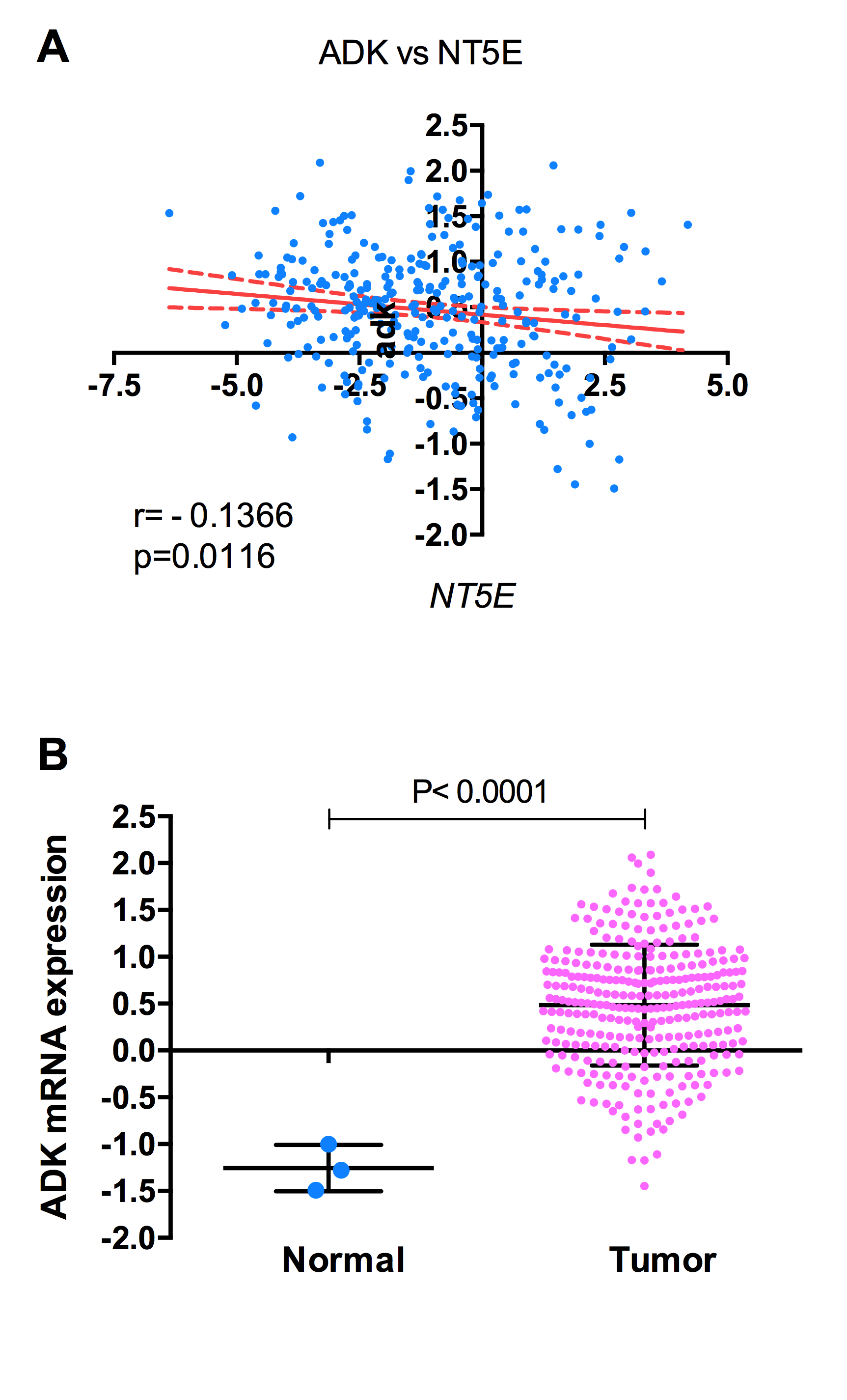

Supplement: Iser et al. supplementary material [file S1462399424000309sup001.zip › Figure S3_ADK.tiff]
